# Two‐Stage Bipolaron Formation in Molecularly Doped Conjugated Polymers

**DOI:** 10.1002/adma.202504357

**Published:** 2025-08-06

**Authors:** Rui Su, Jingshan Chai, Yusen Pei, Yusuf Olanrewaju, Liang Yan, Justin Neu, Jake Mauthe, Katherine Stewart, Somayeh Kashani, Neha Chaturvedi, Stefan Nikodemski, Jarrett H. Vella, Aram Amassian, David S. Ginger, Ji‐Seon Kim, Harald Ade, Wei You, Franky So

**Affiliations:** ^1^ Department of Materials Science and Engineering North Carolina State University Raleigh NC 27695 USA; ^2^ Organic and Carbon Electronics Laboratories (ORaCEL) North Carolina State University Raleigh NC 27695 USA; ^3^ Department of Fiber and Polymer Science Wilson College of Textiles North Carolina State University Raleigh NC 27606 USA; ^4^ Department of Chemistry University of North Carolina at Chapel Hill Chapel Hill NC 27599 USA; ^5^ Department of Physics and Centre for Processable Electronics Imperial College London Blackett Laboratory London SW7 2AZ UK; ^6^ Department of Physics North Carolina State University Raleigh NC 27695 USA; ^7^ Sensors Directorate Air Force Research Laboratory Wright‐Patterson Air Force Base (AFB) Dayton OH 45433 USA; ^8^ Department of Chemistry University of Washington Seattle WA 98195 USA; ^9^ Department of Chemistry University of Oxford Oxford OX1 3TA UK

**Keywords:** bipolarons, conjugated polymers, dip doping, double doping, glycol sidechains

## Abstract

The formation and dynamics of bipolarons are crucial in determining the electrical properties of molecularly doped conjugated polymers. Traditionally, bipolarons are known to form at very high doping levels through the combination of two adjacent polarons, a process that is generally accompanied by structural disorder and impaired carrier transport. Here, it is demonstrated that bipolaron formation can occur at both the early stage with low doping levels and the late stage with high doping levels in 2,3,5,6‐tetrafluoro‐7,7,8,8‐tetracyanoquinodimethane (F_4_TCNQ) dip‐doped conjugated polymer films with glycol sidechains. Bipolaron formation at the early stage is discovered to be mainly associated with double doping, which is uncommon in conventional doped polymer systems. In contrast, bipolaron formation at the late stage is dominated by combining two polarons. Furthermore, these bipolarons are observed to behave differently: early‐stage bipolarons generated through double doping enhance both the molecular ordering and carrier transport, whereas late‐stage bipolarons resulting from polaron combination occur alongside detrimental effects in structural and transport properties. These findings provide new insights into the mechanisms of bipolaron formation across different doping levels and underscore the potential for optimizing doping strategies. A deeper understanding of bipolarons can guide the design of next‐generation molecularly doped conjugated polymers with improved performance.

## Graphical abstract

This work investigates bipolaron formation in 2,3,5,6‐tetrafluoro‐7,7,8,8‐tetracyanoquinodimethane (F_4_TCNQ)‐doped conjugated polymer P(g_3_2T‐T) films with glycol sidechains at two stages of both low and high doping levels. At the early stage, bipolarons mainly form from double doping, enhancing structural ordering, and carrier transport. In contrast, late‐stage bipolarons, mostly generated by polaron combination, exhibit adverse effects on those properties.

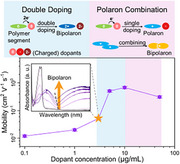

## 1 Introduction

Molecular doping in conjugated polymers (CPs) generates charge carriers, significantly enhancing the electrical conductivity of these materials. Such doped CPs have garnered tremendous interest due to their potential applications in advanced electronic devices, including organic light‐emitting diodes,^[^ ^1^, ^2^ ^]^ thin‐film transistors,^[^ ^3^, ^4^ ^]^ and organic photovoltaics.^[^ ^5^, ^6^ ^]^ Achieving high performance in these devices and advancing their practical applications necessitates a comprehensive understanding of charge carrier formation and transport dynamics in doped CP systems.

In molecularly doped CPs, doping involves either the removal (p‐type) of an electron from or addition (n‐type) of an electron to a neutral polymer segment, creating a single charge carrier known as a polaron, consisting of the charge itself and its associated lattice distortion.^[^ ^7^, ^8^, ^9^, ^10^ ^]^ Upon further doping, an additional electron can be removed or added, resulting in the formation of a bipolaron, a charge carrier bearing two charges along with their lattice distortion.^[^ ^7^, ^11^ ^]^ The understanding of polarons and bipolarons in doped CPs has been evolving over the years. Early investigations have revealed that polarons are the primary charge carrier species in most polymers.^[^ ^11^, ^12^, ^13^, ^14^, ^15^, ^16^ ^]^ In particular, Furukawa and colleagues have demonstrated that the formation of polarons in polymers such as poly(3‐hexylthiophene) (P3HT)^[^ ^17^ ^]^ and poly(2,5‐bis(3‐hexadecylthiophen‐2‐yl)thieno[3,2‐b]thiophene) (PBTTT‐C_16_)^[^ ^18^ ^]^ leads to enhanced carrier mobility and conductivity upon doping. When doping levels become very high, polarons can combine to form bipolarons,^[^ ^16^, ^19^, ^20^, ^21^, ^22^, ^23^ ^]^ which have almost always been linked to reduced mobility and conductivity associated with structural disorder until now.^[^ ^17^, ^18^, ^24^, ^25^ ^]^ Furthermore, previous studies have shown that the conjugated segment length within a polymer chain influences charge carrier formation. Specifically, longer conjugated segments are more likely to promote bipolaron formation, while shorter segments favor polaron formation.^[^ ^26^ ^]^ In short, the essence of charge carriers and their transport in doped CPs are strongly dependent on the material systems and doping levels. However, specific types of charge carriers, their formation mechanisms, and associated properties are not well understood, highlighting the need for further investigation.

Polymers functionalized with oligoethylene glycol sidechains have recently attracted considerable attention due to their enhanced hydrophilicity, polarity, and ion conductivity,^[^ ^27^, ^28^ ^]^ which are beneficial for doping. These materials thus demonstrate excellent compatibility with common dopants such as 2,3,5,6‐tetrafluoro‐7,7,8,8‐tetracyanoquinodimethane (F_4_TCNQ), a molecule with a deep LUMO energy level. Systems employing these polymers have achieved high doping efficiencies with enhanced electrical conductivity, suggesting promising avenues for device applications.^[^ ^29^, ^30^, ^31^, ^32^, ^33^ ^]^

Beyond material selection, the processing technique is also crucial in achieving effective molecular doping. Two primary doping methods, pre‐doping and post‐doping, are widely employed. Pre‐doping, often referred to as mixed doping, involves prior mixing of a polymer and a dopant, enabling precise control over their respective quantities.^[^ ^34^, ^35^, ^36^ ^]^ However, finding a suitable solvent that guarantees the solubility and miscibility of both polymers and dopants can be challenging and may introduce potential structural distortion.^[^ ^34^, ^37^ ^]^ Alternatively, post‐doping methods, including dip doping and sequential doping, are also frequently employed. These techniques introduce dopants into an existing polymer structure, thereby preserving the integrity of the polymer microstructure with minimal structural disruption.^[^ ^38^, ^39^ ^]^

Herein, we demonstrate a film doping system of a thiophene polymer with triethylene glycol sidechains, P(g_3_2T‐T), dip‐doped with different concentrations of F_4_TCNQ. By examining the optical absorption and spin density as a function of dopant concentration, we observe the formation of bipolarons occurs not only at high doping levels (>5 µg mL^−1^) but also unexpectedly at low doping levels (≤5 µg mL^−1^). We designate 5 µg mL^−1^ as the threshold dopant concentration, as beyond this threshold, the π−π* absorption bands of the polymer are completely bleached. Accordingly, we define the doping levels achieved with dopant concentrations above 5 µg mL^−1^ as “high” and those at or below this threshold as “low” in our system. A quantitative analysis comparing the charge carrier concentration with the spin density validates that bipolarons are the dominant charge carriers across all the doping levels. Additionally, energy level alignment and dopant charge state evolution reveal that double doping dominates at low doping levels and gradually transitions to single doping at high doping levels. By probing the conformational changes of the polymer backbone upon doping and contrasting the effects of F_4_TCNQ doping in P(g_3_2T‐T) with those of a weaker dopant, F_2_TCNQ, we discover that the early‐stage bipolaron formation at low doping levels (≤5 µg mL^−1^) in the F_4_TCNQ‐doped system is mainly associated with double doping. The formed bipolarons result in significantly enhanced charge transport, as shown by the increased carrier mobility and conductivity, setting this stage apart from the late stage with high doping levels (>5 µg mL^−1^), where bipolarons form through polaron combination via single doping, leading to impeded charge transport. Microstructural analysis further demonstrates the difference in the structural ordering between these two stages, which can be correlated with the different mechanisms of bipolaron formation at each stage. These findings shed light on the pathways of bipolaron formation, offering new insights into material design strategies for doped CPs.

## 2 Results and Discussion

**Figure 1:**
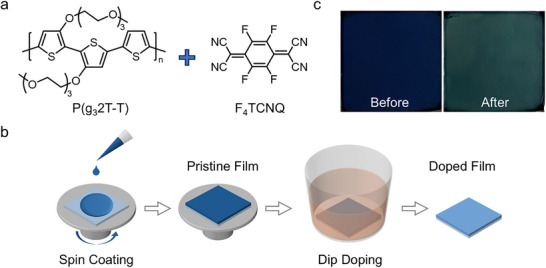
a) Chemical structures of conjugated polymer P(g_3_2T‐T) and the dopant F_4_TCNQ. b) Schematic of dip doping process used to achieve doped films. c) As‐cast pristine and highly doped films (with 50 µg mL^−1^ of F_4_TCNQ).

We employed the dip doping method to dope as‐cast P(g_3_2T‐T) films with F_4_TCNQ, as illustrated in **Figure** **1**a,b. The detailed procedure is provided in the Experimental Section. Dip doping was chosen for its ease of processing and minimal dopant‐induced disorder, as it relies on the diffusion of dopants into pre‐fabricated thin films of CPs, making it capable of achieving a continuous range of doping from low to high.^[^ ^39^ ^]^ This approach enables us to investigate the correlation between the film morphology, optical properties, and carrier transport properties. First, pristine P(g_3_2T‐T) films with a thickness of ≈115 nm were fabricated, exhibiting a uniform, fully covered surface with a glossy appearance. These films were subsequently dip‐doped with F_4_TCNQ at concentrations ranging from 0.1 to 50 µg mL^−1^. A noticeable color change from dark blue to yellowish‐green throughout the entire film indicates successful doping, as shown in Figure 1c. Post‐doping, the films retained their surface uniformity, with thicknesses increasing to ≈120−150 nm. The success of doping was further confirmed by UV‐Vis‐NIR absorbance spectra of both undoped and doped films.

**Figure 2:**
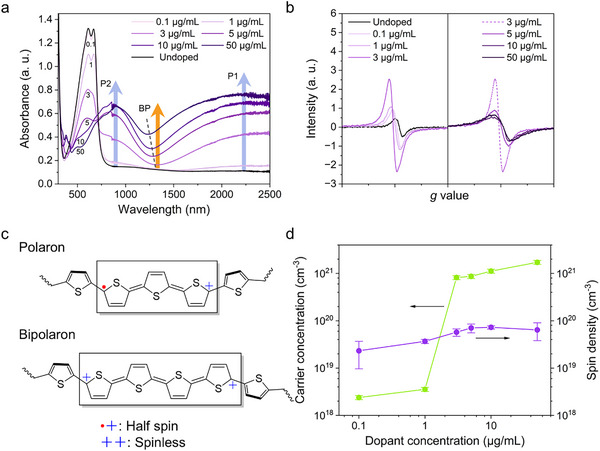
a) UV‐Vis‐NIR absorbance spectra of F_4_TCNQ‐doped P(g_3_2T‐T) films with increasing doping concentrations, highlighting the polaron (P) and bipolaron (BP) absorption features. The grey dotted line marks the central position of the saddle‐like feature as the dopant concentration increases. b) Volume‐normalized electron paramagnetic resonance (EPR) spectra of P(g_3_2T‐T) films doped with varying F_4_TCNQ concentrations. Left: 0−3 µg mL^−1^, Right: 3−50 µg mL^−1^. The dotted line in the right panel at 3 µg mL^−1^ represents data that is repeated from the left panel. c) Schematic of polaron with half spin and spinless bipolaron structures formed along the polythiophene molecular chain. d) Evolution of charge carrier concentration and spin density in P(g_3_2T‐T) films as a function of F_4_TCNQ concentration. Carrier concentrations were measured from alternating‐current (AC) Hall measurements, and spin densities were calculated from the double integral of the normalized EPR spectra. Error bars for the carrier concentrations reflect the measurement uncertainty associated with the AC Hall setup, primarily arising from the systematic phase error of the lock‐in system typically observed in conjugated polymers. Error bars for the spin densities represent the standard deviation from three independent EPR measurements (see Table S1, Supporting Information).

As shown in **Figure** **2**a, the undoped P(g_3_2T‐T) film exhibits a primary absorption band at ≈610 nm, attributed to the π−π* transition of its conjugated backbone.^[^ ^40^ ^]^ Upon doping F_4_TCNQ at concentrations ranging from 0.1 to 50 µg mL^−1^, the absorbance of this π−π* peak decreases, with pronounced bleaching observed. Here, we classify the dopant concentrations at which the π−π* peak remains visible in the UV‐Vis‐NIR absorbance spectra as “low” doping levels (≤ 5 µg mL^−1^), and those at which the π−π* peak has disappeared as “high” doping levels (>5 µg mL^−1^).^[^ ^11^ ^]^ The bleaching fraction increases rapidly at low doping levels, gradually levels off at high doping levels (reaching 72% at 10 µg mL^−1^ of F_4_TCNQ), and eventually plateaus (Figure S1 and Note S2, Supporting Information), indicating the dip‐doping method employed is effective. In addition, for films with dopant concentrations of 10 and 50 µg mL^−1^, a small peak appears at ≈382 nm, corresponding to neutral F_4_TCNQ.^[^ ^32^ ^]^ This neutral signal further confirms that the polymer has been doped at high levels, where the excess dopants are not ionized further.

Moreover, doping leads to the formation of polaron bands, observable as two features at ≈2500 nm (P1) and ≈875 nm (P2) in the UV‐Vis‐NIR absorbance spectra, corresponding to the transitions from the polymer valence band to the lower polaron state and between the lower and upper polaron states within the polymer bandgap, respectively.^[^ ^7^ ^]^ As the dopant concentration increases, these polaron bands exhibit a slight initial increase (0.1–1 µg mL^−1^), followed by a significant rise in absorbance, as expected. Notably, an additional saddle‐like feature^[^ ^16^, ^41^, ^42^ ^]^ emerges at ≈1250 nm (BP) when the dopant concentration reaches 3 µg mL^−1^. This feature intensifies significantly with higher dopant concentrations, accompanied by a noticeable blue shift of its central position. Considering the bipolaron band associated with the transition from the polymer valence band to the bipolaron state within the polaron states,^[^ ^16^, ^43^ ^]^ the observed saddle‐like feature and its evolution suggest the formation and subsequent increase of the bipolaron population,^[^ ^7^, ^24^, ^44^, ^45^, ^46^ ^]^ as evidenced by the fitted UV‐Vis‐NIR spectra (Figure S2, Supporting Information).

To confirm the nature of polarons and bipolarons generated in our doped system, electron paramagnetic resonance (EPR) measurements were conducted on films with equivalent F_4_TCNQ concentrations as those used in the UV‐Vis‐NIR absorbance spectra (Figure 2b). Compared to the low EPR peak intensity of the undoped sample, the EPR peak intensity of the doped films increases gradually as the dopant concentration increases from 0.1 to 3 µg mL^−1^. However, when the dopant concentration exceeds 3 µg mL^−1^, the intensity begins to decrease, and the peak broadens. Figure 2c schematically shows the polaron and bipolaron structures in our system. The polaron carries a positive charge with a half spin^[^ ^7^, ^47^, ^48^ ^]^ localized along the thiophene rings, which can be detected by EPR measurements. In contrast, the bipolaron, which bears two positive charges, exhibits paired spins and thus produces no EPR signal.^[^ ^15^ ^]^ Therefore, the observed increase in the EPR peak intensity below 3 µg mL^−1^ can be attributed to the formation of polarons, with more polarons yielding additional unpaired spins. Beyond 3 µg mL^−1^, the subsequent decrease and broadening in the EPR signal at higher dopant concentrations suggest the gradual formation of bipolarons, in agreement with the UV‐Vis‐NIR results.

Furthermore, to quantify the bipolarons formed in our system, we performed alternating‐current (AC) Hall measurements to determine the carrier concentrations of all F_4_TCNQ‐doped P(g_3_2T‐T) samples (Figure 2d). These measurements provide the total mobile carrier concentration, including contributions from both polarons and bipolarons. Upon initial doping, the carrier concentration slightly increases from 2.4 × 10^18^ cm^−3^ at 0.1 µg mL^−1^ to 3.6 × 10^18^ cm^−3^ at 1 µg mL^−1^ of F_4_TCNQ. At 3 µg mL^−1^, the carrier concentration sharply increases to 8.2 × 10^20^ cm^−3^ by two orders of magnitude. A significantly reduced activation energy (*E*_a_) of carriers at this concentration fitted from temperature‐dependent AC Hall measurements (Figures S3 and S4 and Note S3, Supporting Information) corroborates the observed increase in carrier concentration. As the dopant concentration increases further, the carrier concentration continues to rise, reaching 1.7 × 10^21^ cm^−3^. Comparing carrier concentrations with spin densities obtained from the double integral of the EPR spectra^[^ ^49^ ^]^ for each doped sample (Figure 2d), the spin densities from 0.1 to 1 µg mL^−1^ are on the order of 10^19^ cm^−3^, exceeding the carrier concentrations by an order of magnitude due to limited mobile carriers in this region.^[^ ^50^ ^]^ Beyond 1 µg mL^−1^, the pronounced increase in carrier concentration surpasses the spin density by over an order of magnitude. Given that the onset of bipolaron formation occurs at 3 µg mL^−1^, this signifies the formation of substantial bipolarons.

We then estimated the bipolaron percentage, as the ratio of bipolaron number to the total counts of bipolarons and polarons, using the carrier concentration from AC Hall measurements and the spin density from EPR measurements, as detailed in Note S4 (Supporting Information). As listed in Table S2 (Supporting Information), the bipolaron percentages are over 85% for all samples, indicating that bipolarons are the dominant carriers in the studied system. It should be noted that for dopant concentrations of 10 and 50 µg mL^−1^, the UV‐Vis‐NIR absorbance spectra (Figure 2a) show the characteristic absorption peaks of F_4_TCNQ^−^ at 765 and 862 nm,^[^ ^33^ ^]^ suggesting that the bipolaron percentages are likely underestimated in these cases, as the polaron numbers may be overestimated due to the additional spin contribution from F_4_TCNQ^−^. In addition, a small drop in bipolaron percentage at 5 µg mL^−1^ (Table S2, Supporting Information) combined with the peak spin density within the doping range (Figure 2d) suggests that there may be a transition in the bipolaron formation mechanisms below and above this concentration, which will be discussed later.

**Figure 3:**
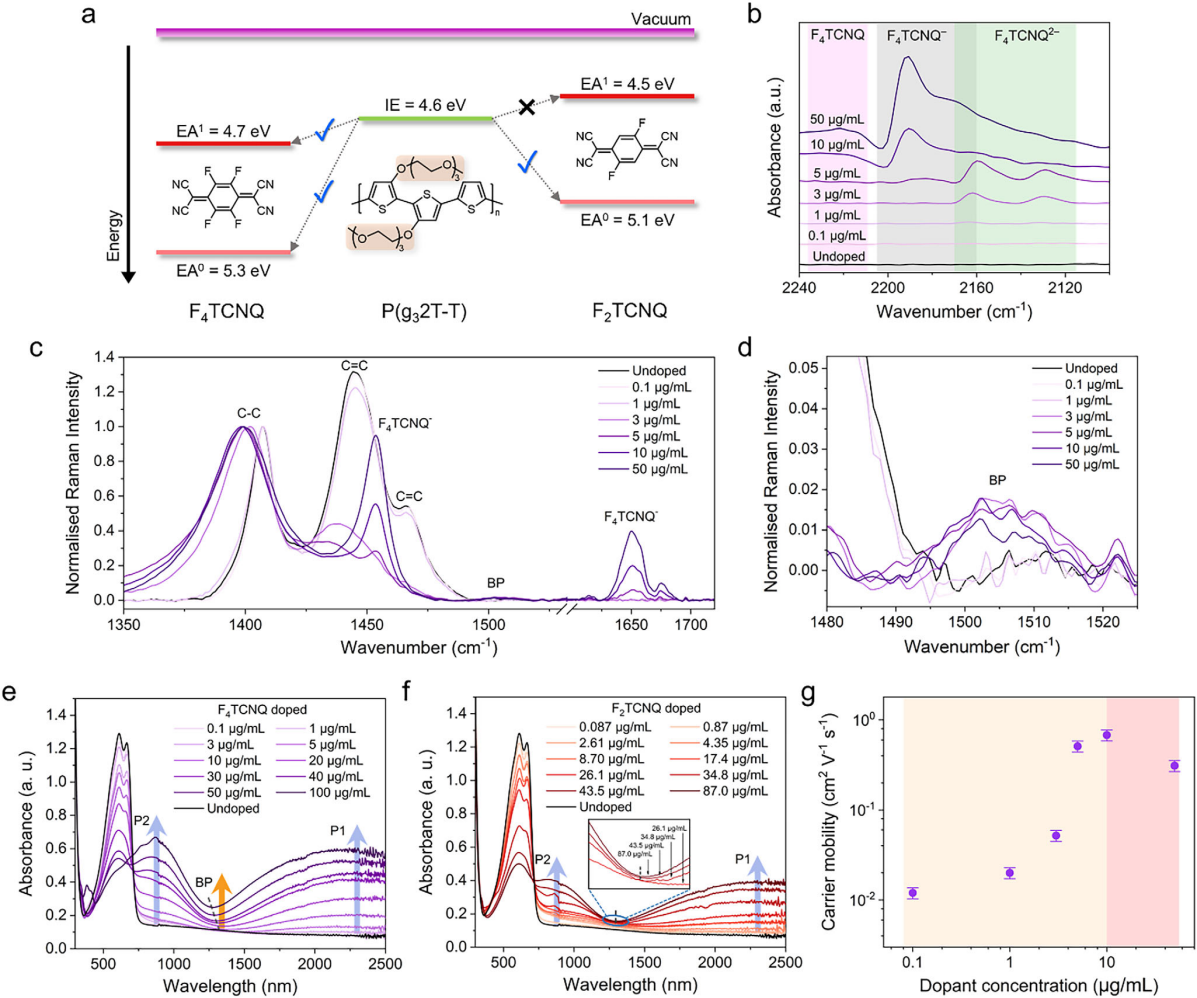
a) Energy diagram of P(g_3_2T‐T) and dopants measured by cyclic voltammetry (CV), showing ionization energy (IE) and electron affinity (EA), respectively. Arrows with crosses or ticks indicate whether electron transport is energetically forbidden or allowed. b) Fourier transform infrared (FTIR) absorbance spectra of the cyano (CN) stretch vibrations of neutral F_4_TCNQ (pink region), F_4_TCNQ^−^ (grey region), and F_4_TCNQ^2−^ (green region) in dip‐doped samples with varying dopant concentrations. c) Resonance Raman spectra of F_4_TCNQ dip‐doped P(g_3_2T‐T) films acquired with 785 nm excitation. d) Magnified view of the 1480–1525 cm^−1^ region highlighting the bipolaron peak at 1505 cm^−1^. e,f) UV‐Vis‐NIR absorbance spectra of P(g_3_2T‐T) films doped with e) F_4_TCNQ and f) F_2_TCNQ at various concentrations using the sequential doping method. P: polaron, BP: bipolaron. Inset: Enlarged view of the region ≈1250 nm from 26.1 to 87 µg mL^−1^ of F_2_TCNQ concentration. The center of the saddle‐like feature with increasing dopant concentrations is denoted by the grey dotted line. g) Charge carrier mobility of dip‐doped P(g_3_2T‐T) samples as a function of F_4_TCNQ concentration, determined through AC Hall measurements with associated uncertainties. Two background colors are applied to differentiate the two stages of bipolaron formation at low and high doping levels.

It is noteworthy that such an early‐stage formation of bipolarons at low doping levels (≤5 µg mL^−1^) has been rarely reported. In general, bipolarons typically emerge only at high doping concentrations through the combination of two nearby polarons, as previously mentioned. To elucidate the mechanism driving bipolaron formation at low doping levels, we considered the energy level structures of both the polymer and dopants. The ionization energy (IE) of P(g_3_2T‐T) is determined to be 4.6 eV by cyclic voltammetry (CV) measurements (Figure S5 and Table S3, Supporting Information), which is notably smaller than that of most conjugated polymers having an IE above 5.0 eV.^[^ ^31^, ^51^, ^52^ ^]^ This reduction in IE can be attributed to the glycol sidechains linked via an oxygen atom to thiophene in P(g_3_2T‐T), which enhance the electron density on the thiophene rings due to their strong electron‐donating ability^[^ ^27^ ^]^ and high flexibility,^[^ ^28^ ^]^ thereby raising the polymer's energy level, as shown in **Figure** **3**a. In addition, the electron affinities (EAs) of neutral F_4_TCNQ (EA^0^) and F_4_TCNQ^−^ (EA^1^) from the CV measurements (Figure S6 and Table S3, Supporting Information) are 5.3 and 4.7 eV, respectively. We observed that EA^1^ value of F_4_TCNQ^−^ remains slightly larger than the IE value of P(g_3_2T‐T), alongside the much larger EA^0^ value of F_4_TCNQ. These deeper energy levels of the dopant suggest the possibility of further reduction of F_4_TCNQ^−^ to F_4_TCNQ^2^^−^ after P(g_3_2T‐T) undergoes oxidation by F_4_TCNQ during doping, a process known as “double doping”.^[^ ^32^ ^]^ In this mechanism, a single dopant molecule facilitates two electrons transfer with the polymer, which has been observed in polymers containing similar glycol sidechains.^[^ ^32^, ^33^, ^53^, ^54^, ^55^ ^]^ Impressively, in the P(g_4_2T‐TT) polymer reported by Keifer et al., the double‐doping effect pushes the ionization efficiency approaching 200% at low dopant concentrations below 10 mol%.^[^ ^32^ ^]^ Moreover, at low doping levels (≤5 µg mL^−1^), despite the clear emergence of bipolaron features at 3–5 µg mL^−1^ (Figure 2a), the absence of the characteristic F_4_TCNQ^−^ absorption peaks (as aforementioned) verifies that F_4_TCNQ is mostly ionized to F_4_TCNQ^2^^−^, consistent with the double‐doping mechanism. To further confirm the charged states of F_4_TCNQ, we performed Fourier transform infrared (FTIR) measurements on these films used in the UV‐Vis‐NIR analysis. As shown in Figure 3b, the FTIR absorbance spectra in the 2100–2240 cm^−1^ range reveal distinct cyano (CN) stretch vibrations corresponding to F_4_TCNQ species.^[^ ^32^, ^56^ ^]^ F_4_TCNQ^2^^−^ (green region) exhibits two peaks at ≈2160 and ≈2130 cm^−1^, F_4_TCNQ^−^ (grey region) shows a doublet with a main peak at ≈2190 cm^−1^ and a broad shoulder at ≈2170 cm^−1^, and neutral F_4_TCNQ (pink region) displays a single peak centered at ≈2220 cm^−1^. We observed F_4_TCNQ^2^^−^ features emerging at concentrations as low as 1 µg mL^−1^ and increasing, peaking at 5 µg mL^−1^ where a faint F_4_TCNQ^−^ signal first appears. With further increase in dopant concentration (≥10 µg mL^−1^), the F_4_TCNQ^−^ peak becomes more pronounced, and the neutral F_4_TCNQ feature develops. These trends are more apparent by tracking the signal intensities versus dopant concentration in Figure S7 (Supporting Information). F_4_TCNQ^2−^ grows rapidly and dominates at low doping levels (≤5 µg mL^−1^), while F_4_TCNQ^−^ and neutral F_4_TCNQ remain negligible until high doping levels (>5 µg mL^−1^) are reached, where their signals begin to strengthen. These results clarify that at low doping levels, charge transfer primarily occurs through double doping, whereas at high doping levels, it gradually shifts toward single‐doping dominance. Together with the favorable energy alignment between the polymer and dopant, the lack of F_4_TCNQ^−^ signals in the UV‐Vis‐NIR absorbance spectra, and the clear FTIR features, we propose that bipolaron formation at this early stage (≤5 µg mL^−1^) is mainly driven by the double‐doping process.

To provide direct evidence for this double‐doping‐induced bipolaron formation, we employed resonance Raman spectroscopy to probe the associated conformational changes in the polymer backbone upon doping. The Raman spectra of the F_4_TCNQ dip‐doped P(g_3_2T‐T) samples, presented in Figure 3c, were acquired with an excitation at 785 nm, which is resonant with the polaronic band, the tail of the bipolaron band, and the F_4_TCNQ^−^. For the undoped sample, the two peaks observed in the 1400–1450 cm^−1^ range correspond to the C─C and C═C vibrations of the P(g_3_2T‐T) backbone. Upon doping, a characteristic shift in the peak positions and a decrease in the C═C:C─C intensity ratio obviously appear at 3 µg mL^−1^ and then continue with increasing doping levels. To ensure reliable comparison, all spectra were normalized to the C─C vibronic mode, which remains relatively insensitive to doping. These spectral changes demonstrate an efficient transition of the P(g_3_2T‐T) conjugated backbone from a benzoidal to a more quinoidal‐like structure^[^ ^25^, ^57^, ^58^ ^]^ starting at 3 µg mL^−1^, as the polymer backbone rearranges to accommodate the charges upon doping, indicating the formation of polarons/bipolarons (Figure 2c). Additionally, the relative intensity of the F_4_TCNQ^−^ peak at 1651 cm^−1^ only becomes discernible beginning at 5 µg mL^−1^ and increasing sharply at high doping levels. This delayed onset aligns with the FTIR results (Figure 3b). Notably, F_4_TCNQ^2^^−^ is not detected due to the negligible resonance. Therefore, the absence of the F_4_TCNQ^−^ Raman signal at low doping levels unlike common observations,^[^ ^59^, ^60^ ^]^ particularly at 3 µg mL^−1^ where significant quenching of the P(g_3_2T‐T) backbone signal (C═C mode) is already evident—suggests the presence of an alternative doping mechanism in this doping range, namely double doping. Furthermore, the bipolaron‐specific peak^[^ ^25^, ^61^ ^]^ centered at 1505 cm^−1^ is highlighted in Figure 3d. Remarkably, this bipolaron peak appears distinctly even at doping levels as low as 3 µg mL^−1^ with pronounced relative intensity. These findings reveal a uniquely high fraction of bipolarons formed through double doping at low doping levels, enabling direct probing of bipolarons prior to the saturation of molecular structural changes in the P(g_3_2T‐T) backbone, which is rarely reported.

To further confirm the correlation between double doping and bipolaron formation, we introduced F_2_TCNQ as a dopant for comparison. With a molecular structure similar to that of F_4_TCNQ, F_2_TCNQ helps to minimize the structural disorder upon doping. However, with two fewer fluorine atoms than F_4_TCNQ, F_2_TCNQ is less oxidative than F_4_TCNQ, as evidenced by the 0.2 eV smaller EA^0^ and EA^1^ values of F_2_TCNQ determined by CV measurements (Figure S6 and Table S3, Supporting Information), as shown in Figure 3a. In this case, F_2_TCNQ can only accept one electron from P(g_3_2T‐T), and the second electron transfer would be forbidden due to the larger IE of P(g_3_2T‐T) compared to the EA^1^ of F_2_TCNQ. Thus, double doping is thermodynamically prohibited, allowing only single doping leading to polaron formation.

We then fabricated P(g_3_2T‐T) thin films doped with varying concentrations of F_4_TCNQ and F_2_TCNQ to verify these findings, ensuring an equivalent molarity at each dopant concentration. The corresponding mass concentrations for the dopants are provided in Table S4 (Supporting Information). It should be pointed out that, due to the solubility issues of P(g_3_2T‐T) films in F_2_TCNQ solutions, all samples in this comparison study were prepared using sequential doping instead, a similar post‐doping approach as dip doping without altering the base structure of polymer films. Further details can be found in the Experimental Section. The UV‐Vis‐NIR absorbance spectra of F_4_TCNQ‐doped P(g_3_2T‐T) films via sequential doping, shown in Figure 3e, exhibit consistent spectral shapes, characteristic peak positions, and trends that are consistent with those obtained through dip doping (Figure 2a), as the F_4_TCNQ concentration increases from 0.1 to 100 µg mL^−1^. This validates the use of sequential doping as an effective alternative in our experiments. As we described earlier, for these samples sequentially doped with F_4_TCNQ, the disappearance of the π−π* peak at the dopant concentration of 100 µg mL^−1^ indicates that the “low” doping levels correspond to the concentrations below 100 µg mL^−1^, while the concentrations at and above that are classified as the “high” doping levels. Specifically, a distinct bipolaron feature (≈1250 nm) emerges at a low dopant concentration of 10 µg mL^−1^, gradually increasing in intensity and blue‐shifting with higher dopant concentrations. In contrast, for the F_2_TCNQ‐doped case (Figure 3f), the saddle‐like feature becomes evident only at concentrations exceeding 34.8 µg mL^−1^ but shows minimal changes at higher dopant concentrations, suggesting the absence of bipolaron formation. Correspondingly, even at the highest tested concentration of 87 µg mL^−1^, the π−π* peak remains, indicating that the F_2_TCNQ‐doped system has not reached the “high” doping level regime that is readily achieved in the case of F_4_TCNQ‐doped system. Instead, all tested concentrations, ranging from 0.087 to 87 µg mL^−1^, fall within the “low” doping levels. Moreover, the F_2_TCNQ^−^ peaks (≈765 and 860 nm)^[^ ^62^ ^]^ are consistently present from 8.7 µg mL^−1^ to higher dopant concentrations. For the concentration of F_2_TCNQ at 43.5 and 87 µg mL^−1^, the relatively weak F_2_TCNQ^−^ peaks (less intense than the F_4_TCNQ^−^ peaks^[^ ^62^ ^]^) may be buried within the overlapping polaron band (P2), making them less apparent. This suggests that only polarons are generated via single doping in the F_2_TCNQ‐doped system, as indicated by the energy level alignment (Figure 3a). However, in the case of F_4_TCNQ doping, no F_4_TCNQ^−^ signals (765 and 862 nm)^[^ ^32^, ^62^ ^]^ are observed at low doping levels (<100 µg mL^−1^) beyond the onset of bipolaron formation at 10 µg mL^−1^, until the transition to the high doping level at 100 µg mL^−1^, at which the neutral F_4_TCNQ peak 382 nm appears. These findings suggest that, at the early stage with low doping levels (<100 µg mL^−1^), bipolarons formed in F_4_TCNQ‐doped P(g_3_2T‐T) mainly arise from double doping. At the late stage with high doping levels (≥100 µg mL^−1^), F_4_TCNQ dopes P(g_3_2T‐T) mostly through single doping, with the observed bipolarons resulting from the combination of polarons.

In addition, as calculated in Table S5 (Supporting Information), the bleaching fraction of the π−π* band is notably higher in F_4_TCNQ‐doped films at low doping levels compared to their F_2_TCNQ counterparts, confirming the occurrence of double doping.^[^ ^32^, ^33^ ^]^ However, this difference reduces markedly when entering high doping levels, indicating that the doping mechanism in the F_4_TCNQ‐doped films is transitioning toward a mechanism similar to that observed for the F_2_TCNQ‐doped cases, that is, single doping. In fact, double doping and single doping are two competing processes, with the dominant mechanism being determined by the relative amounts of the polymer (e.g., P(g_3_2T‐T)) and the dopants (e.g., F_4_TCNQ). At low dopant concentrations, the polymer is in sufficient quantities, enabling extensive interactions with the dopants and thus facilitating double doping provided that the energetics are favorable for double doping. As the dopant concentration increases, the available polymer becomes insufficient to sustain further oxidation, leading to the dominance of single doping. Therefore, the ratio of double doping to single doping decreases with increasing dopant concentrations,^[^ ^32^ ^]^ as evident by the FTIR absorbance spectra (Figure 3b; Figure S7, Supporting Information). This also explains the early‐stage decline in the bipolaron percentage as the dopant concentration increases, as seen in Table S2 (Supporting Information). When entering the late stage, polaron combination primarily contributes to bipolaron formation, leading the bipolaron ratio to rise again (Figure S2, Supporting Information) while simultaneously reducing the spin density as shown in Figure 2d.

To further investigate the charge transport properties associated with bipolarons formed during both the early and late stages, governed by the different mechanisms (vide supra), we measured the carrier mobility and conductivity of F_4_TCNQ‐doped films with dip doping. As shown in Figure 3g, the mobility determined by AC Hall measurements exhibits a sharp increase from 0.02 to 0.52 cm^2^ V^−1^ s^−1^ with the dopant concentration increasing from 1 to 5 µg mL^−1^ preceded by a slight rise from 0.012 to 0.02 cm^2^ V^−1^ s^−1^ over 0.1–1 µg mL^−1^. Subsequently, the mobility undergoes a more modest increase, reaching a maximum of 0.68 cm^2^ V^−1^ s^−1^ at 10 µg mL^−1^ before declining to 0.33 cm^2^ V^−1^ s^−1^ at 50 µg mL^−1^. Consistent results are observed in the space charge limited current (SCLC) and direct‐current (DC) Hall measurements (Figures S8 and S9, Supporting Information). This behavior suggests that bipolarons formed at the early stage (≤5 µg mL^−1^) facilitate enhanced carrier transport. Conversely, at the late stage (>5 µg mL^−1^), bipolaron formation impedes carrier transport, which is consistent with other systems reported previously.^[^ ^17^, ^18^, ^24^, ^25^ ^]^ Similar dopant concentration‐dependent variations in conductivity corresponding to the two‐stage bipolaron formation, are also observed in Figure S10 (Supporting Information).

**Figure 4:**
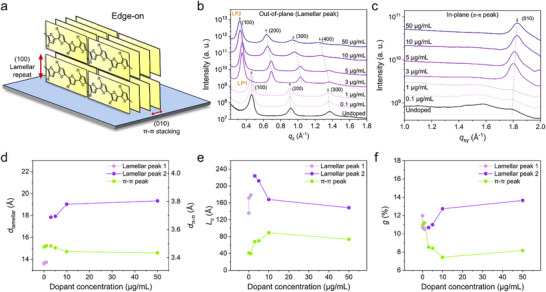
a) Scheme of P(g_3_2T‐T) crystalline structure. 1D grazing incidence wide‐angle X‐ray scattering (GIWAXS) profiles of P(g_3_2T‐T) samples along b) out‐of‐plane and c) in‐plane directions with different dopant concentrations. LP: lamellar peak. Evolution of d) interplanar spacing (d), e) coherence length (*L*_c_), and f) paracrystallinity parameter (*g*) for the lamellar and π−π stacking peaks as a function of dopant concentration, derived from GIWAXS fitting.

Given that carrier transport in doped conjugated polymers is a thermally activated process,^[^ ^63^ ^]^ the measured mobility is determined not only by the doping level but also by the structural disorder introduced upon doping. To further investigate, we characterized the microstructure and local ordering of both the undoped and doped films by employing grazing incidence wide‐angle X‐ray scattering (GIWAXS) measurements. For the undoped P(g_3_2T‐T) film, the lamellar (*h*00) peaks appear in the out‐of‐plane direction, while the π−π peak (010) is observed in the in‐plane direction as shown in the 2D pattern (Figure S11, Supporting Information), indicating a preferred edge‐on orientation (**Figure** **4**a).^[^ ^64^ ^]^ The presence of higher‐order lamellar peaks, including (100), (200), and (300), suggests a highly ordered lamellar structure. Upon introducing a slight concentration of F_4_TCNQ (0.1–1 µg mL^−1^), very small shifts are observed in the positions of the lamellar (≈0.46 Å^−1^) and π−π (≈1.8 Å^−1^) peaks, as seen from the extracted 1D linecuts (Figure 4b,c). However, the weakening of the π−π peak intensity suggests a loss of order in π−π stacking, potentially pointing to the formation of a 2D ordered crystalline structure along the lamellar direction.

As the dopant concentration increases (<10 µg mL^−1^), the lamellar peak shifts to lower *q*‐values (≈0.35 Å^−1^), indicating an increase in lamellar spacing. The emergence of the (400) peak at higher concentrations further supports the enhancement of lamellar ordering. To differentiate between the lamellar peaks at different *q*‐values, we designated the previous higher‐*q* lamellar peaks as lamellar peak 1 (LP1) and the lower‐*q* peaks as lamellar peak 2 (LP2) (Figure 4b). Additionally, the π−π peak shifts to higher *q*‐values (≈1.83 Å^−1^), indicating a decrease in the π−π stacking distance with higher dopant concentrations. These observations suggest the formation of a 3D crystalline structure with dopant molecules residing within the lamellar regions. At dopant concentrations above 10 µg mL^−1^, both the lamellar and π−π peaks broaden and become less distinct, with the (400) lamellar peak nearly disappearing, indicating a reduction in the 3D ordering.

We further analyzed the 1D profiles by fitting the out‐of‐plane (100) lamellar and in‐plane (010) π−π peaks for all films. Figure 4d presents the interplanar spacings (Note S5, Supporting Information), where the LP1 spacing remains at ≈13.7 Å below 1 µg mL^−1^ while the LP2 spacing initially increases due to the presence of the dopant molecules and stabilizes at higher concentrations (≥10 µg mL^−1^). Similarly, the π−π stacking distance shows minimal changes at the very beginning (<1 µg mL^−1^) but decreases as the concentration increases, eventually saturating at higher concentrations (≥10 µg mL^−1^). Two key parameters are then evaluated to examine the structural disorder induced by doping at various levels: the coherence length (*L*_c_) and the paracrystallinity parameter (*g*) (Note S5, Supporting Information). Here, *L*_c_ represents the average size of crystalline domains, and *g* describes the degree of lattice disorder within the imperfect crystal structure. As shown in Figure 4e, *L*_c_ for the lamellar ordering (LP1) initially increases from 136 Å for the undoped film to 179 Å at 1 µg mL^−1^ for the doped film, with unchanged *L*_c_ of the π−π peak, confirming the lamellar stacking of the 2D structure. Further increase in dopant concentration to 3 µg mL^−1^ raises *L*_c_ of the lamellar peak to 223 Å, along with the increased *L*_c_ of the π−π peak from 40 to 68 Å, indicating the onset of stacking along the π−π direction for 3D ordering. Between 3 and 5 µg mL^−1^, a slight decrease in *L*_c_ of LP2, accompanied by a modest increase in that of the π−π peak correspond to enhanced 3D ordering. When the dopant concentration further increases to 10 µg mL^−1^, a decrease in *L*_c_ is observed for LP2, while the π−π peak *L*_c_ still increases to reach a maximum of 89 Å, suggesting that excessive dopants begin to disrupt the lamellar spacing. Beyond 10 µg mL^−1^, the *L*_c_ values of both LP2 and π−π peak decline, indicating that the lattice cannot accommodate further dopants effectively.

The corresponding *g*‐parameter shows an opposite trend (Figure 4f). Specifically, for LP1, *g* decreases from 12% for the undoped film to 10.5% at 1 µg mL^−1^, with *g* of the π−π peak remains ≈11% in this doping range, indicative of the improved 2D crystalline order along the lamellar direction. From 1 to 3 µg mL^−1^, *g* stabilizes for LP2 but decreases for π−π peak, marking the transition from 2D to 3D stacking along the π−π direction. Both an increase in *g* for LP2 and a decrease in the π−π peak between 3 and 5 µg mL^−1^ confirm further improved 3D ordering. When dopant concentration reaches 10 µg mL^−1^, the *g* value of LP2 largely increases to 13.6% while the π−π peak *g*‐parameter decreases to a minimum value of 7.4%, and the results can be attributed to the start of excessive dopant residing into the lamellar spacing. For dopant concentrations exceeding 10 µg mL^−1^, the increases of the *g* values in both LP2 and π−π peak indicate that further growth of crystalline domains being hindered due to substantial disorder from excessive dopant incorporation.

The correlation between induced microstructural changes and the observed change in mobility is closely correlated with the bipolaron formation across the range of doping levels. At the onset of doping (≤1 µg mL^−1^), the charge transport remains largely unaffected due to the limited bipolaron and polaron population. This minimal change is associated with the initially disrupted π−π ordering within the crystalline structure, hindering the charge transport. At low doping levels (3–10 µg mL^−1^), a notable enhancement in mobility emerges, corresponding with the early‐stage bipolaron formation. In this regime, the residence of F_4_TCNQ within the lamellar spacing enhances lamellar and π−π ordering, leading to lamellar spacing expansion and π−π spacing reduction. These structural refinements contribute directly to the observed increase in carrier mobility. However, at high doping levels (≥10 µg mL^−1^), a decline in mobility is noted, concurrent with the late‐stage bipolaron formation and the disrupted growth of crystalline domains at these high dopant concentrations.

**Figure 5:**
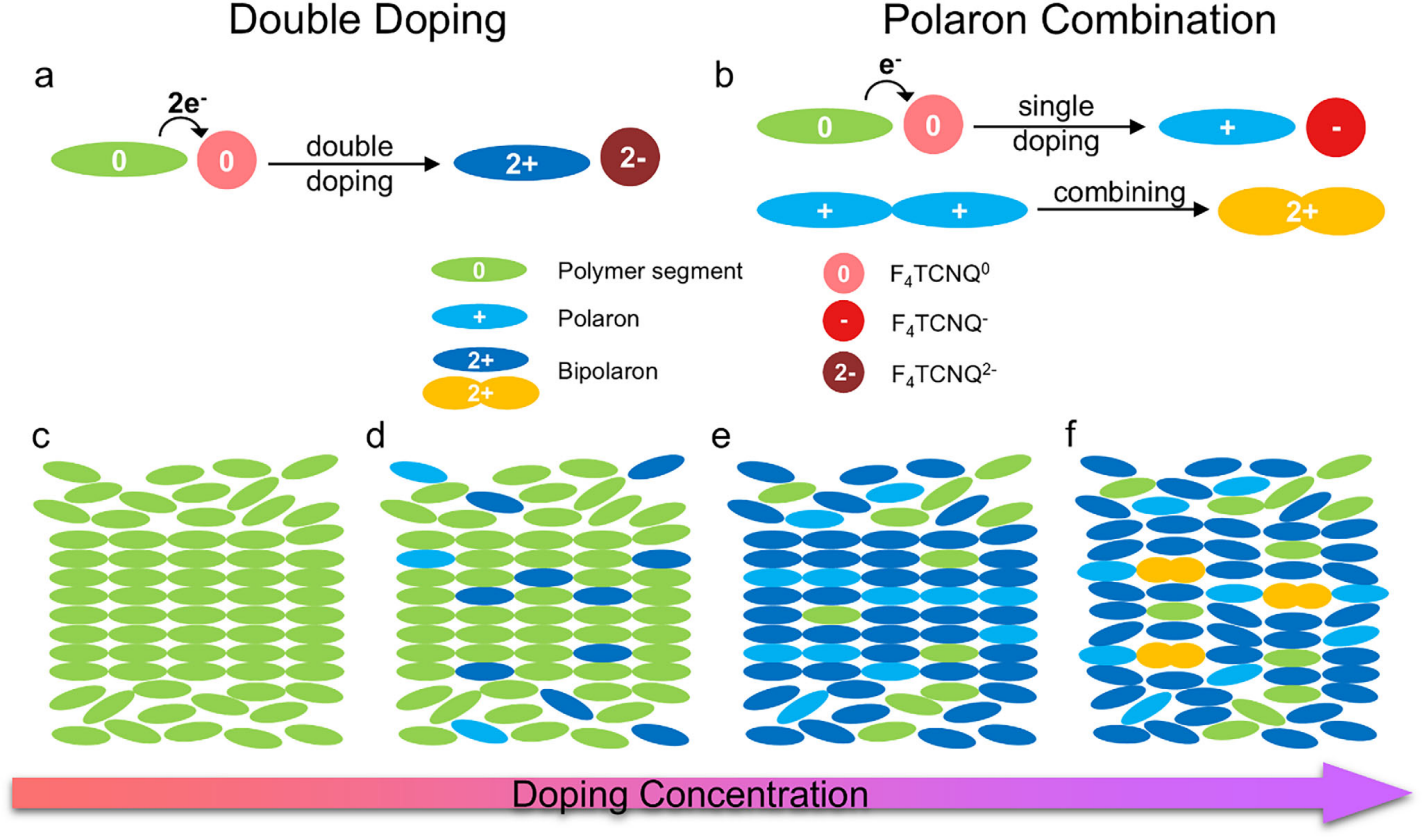
a,b) Schematics of bipolaron formation mechanisms in P(g_3_2T‐T) through a) double doping and b) polaron combination as the concentration of F_4_TCNQ increases. c–f) Diagrams illustrating the evolution of charge carrier formation in P(g_3_2T‐T) across the overall doping range of F_4_TCNQ.

Based on the findings above, we propose a picture of the doping process in the studied system, elucidating the bipolaron formation and its effects across a large range of doping levels. Essentially, bipolaron formation in F_4_TCNQ‐doped P(g_3_2T‐T) films is via two mechanisms:

1. Double doping at early stage: A neutral F_4_TCNQ (F_4_TCNQ^0^) molecule accepts two electrons from a neutral polymer segment, forming an F_4_TCNQ^2−^, directly generating a bipolaron through the double‐doping process (**Figure** **5**a).
2. Polaron combination at late stage: A neutral F_4_TCNQ (F_4_TCNQ^0^) accepts one electron from a neutral polymer segment, creating an F_4_TCNQ^−^ and a polaron, following the common single‐doping process. Two polarons in close proximity subsequently combine to form a bipolaron (Figure 5b).

Initially, upon introducing dopants into the undoped polymer matrix (Figure 5c), only a limited number of polymer segments become doped (Figure 5d), mostly generating bipolarons through double doping, which enhance the structural ordering and transport properties of the polymer. Along with the formation of bipolarons, a small number of polarons are also formed. As the doping level increases (Figure 5e), bipolarons are generated in a large number as well as polarons. Within this range, the molecular ordering continues to increase as planarity increases, leading to enhanced charge transport properties. At the same time, the dominant doping mechanism gradually shifts from double doping to single doping, resulting in a decrease of the bipolaron to polaron ratio. As doping reaches high levels, single doping becomes the dominant mechanism, producing a large population of polarons. The resulting high polaron concentration brings individual polarons closer to each other, making them combine easily into bipolarons, leading to further increase in the bipolaron population (Figure 5f). However, this combination disrupts the crystalline/molecular order, impeding the carrier transport properties.

## 3 Conclusion

This study elucidates a two‐stage bipolaron formation in the F_4_TCNQ‐doped P(g_3_2T‐T) film system. Through a comprehensive analysis of data from various characterization techniques, we demonstrated that bipolarons not only form at high doping levels but also form at low doping levels where bipolaron formation has typically not been observed. Consequently, bipolarons serve as the dominant carriers in the studied system. We also identified the doping mechanism transitions from double doping at low doping levels to single doping at high doping levels. Comparative investigations between F_4_TCNQ and F_2_TCNQ doped P(g_3_2T‐T) films reveal that double doping is mainly responsible for bipolaron formation at the early stage, while bipolarons are mostly formed by polaron combination through single doping at the late stage. Notably, we found that bipolarons formed at the early and late stages exhibit distinct behaviors in terms of mobility and conductivity, with the early‐stage bipolarons significantly enhancing the transport properties, whereas the late‐stage bipolarons lead to structural disorder and impede carrier transport. Furthermore, GIWAXS results highlight the improved structural ordering at the early stage, which subsequently deteriorates at the late stage. Our work provides insights into the mechanism of bipolaron formation in conjugated polymers with glycol sidechains and offers valuable guidelines for material selection and design aimed at optimizing charge carrier dynamics through doping in molecularly doped conjugated polymers.

## 4 Experimental Section

### Materials

Poly[5,5′‐(3,3‐bis(2‐(2‐(2‐methoxyethoxy)ethoxy)ethoxy)‐2,2′‐bithiophene)‐alt‐2,5‐thiophene] (P(g_3_2T‐T)) was synthesized following the procedures outlined in Note S1 (Supporting Information). 2,3,5,6‐tetrafluoro‐7,7,8,8‐tetracyanoquinodimethane (F_4_TCNQ, ≥99.8%) was purchased from e‐Ray Optoelectronics Technology. 2,5‐

difluoro‐7,7,8,8‐tetracyanoquinodimethane (F_2_TCNQ, ≥99%) was purchased from TCI Chemicals. Chloroform (CHCl_3_, ≥99.8%) and acetonitrile (CH_3_CN, ≥99.95%) were purchased from Sigma–Aldrich and Fisher Scientific, respectively. All chemicals were used as received without further purification.

### Sample Preparation—Substrate

Glass substrates coated with indium‐tin oxide (ITO) and silicon substrates were sequentially cleaned by sonication in acetone and isopropyl alcohol for 20 min each. The cleaned glass substrates were dried with nitrogen gas, followed by UV‐ozone treatment to remove any remaining organic residues.

### Sample Preparation—P(g_3_2T‐T) Film Preparation

To prepare P(g_3_2T‐T) films, the polymer was first dissolved in CHCl_3_ (20 mg mL^−1^) and stirred the solution overnight at 55 °C in a nitrogen‐filled glovebox. The solution was then spin‐coated onto cleaned glass substrates at 2500 rpm for 45 s.

### Sample Preparation—Dip Doping of P(g_3_2T‐T) Films with F_4_TCNQ

For dip doping, F_4_TCNQ was dissolved in CH_3_CN (1 mg mL^−1^) at 60 °C and diluted to create solutions of varying concentrations (0.1, 1, 3, 5, 10, 50 µg mL^−1^). P(g_3_2T‐T) films were then immersed in these solutions for 10 s each, followed by drying inside a N_2_‐filled glovebox without annealing.

### Sample Preparation—Sequential Doping of P(g_3_2T‐T) Films with F_4_TCNQ and F_2_TCNQ

Dopant solutions of F_4_TCNQ and F_2_TCNQ were prepared by dissolving the respective materials in acetonitrile at initial concentrations of 100 and 87 µg mL^−1^, respectively, at 60 °C. These stock solutions were then diluted to a range of concentrations from 0.1 to 50 µg mL^−1^ for F_4_TCNQ, while maintaining molar equivalence with F_2_TCNQ concentrations, which ranged from 0.087 to 43.5 µg mL^−1^ (Table S4, Supporting Information).To dope the pre‐prepared P(g_3_2T‐T) films, 40 µL of the F_4_TCNQ solution, at the desired concentration, was deposited onto the polymer film and allowed to dry in a N_2_ environment without annealing. The same procedure was followed for doping P(g_3_2T‐T) films with F_2_TCNQ.

### Characterizations—Thickness Measurements

The thickness of all thin films was measured using a P‐7 stylus profiler (KLA Tencor, Milpitas, CA).

### Characterizations—UV‐Vis‐NIR Absorption Spectroscopy

Absorption measurements of the polymer thin films deposited on glass substrates were performed with a PerkinElmer Lambda 750 UV/VIS spectrophotometer.

### Characterizations—Electron Paramagnetic Resonance (EPR) Spectroscopy

EPR measurements were performed using a JEOL X‐Band EPR spectrometer. Samples were prepared by spin‐coating onto uncoated PET substrates cut to a size of 1.5 cm × 0.3 cm. To minimize exposure to air, the samples were then loaded into NMR tubes and sealed within a glovebox. Spin density was calculated by double integration of the EPR curves relative to a reference sample of TEMPOL (0.05 mg, 98%, *M*_w_ = 172.24).

### Characterizations—Alternating‐Current (AC) Hall Measurements

Charge carrier concentration, mobility, and conductivity were measured using the Lakeshore 84030 AC Hall System, applying the van der Pauw four‐point probe technique. Borosilicate substrates were fabricated into a standard van der Pauw configuration with 5 mm separation by sputtering 20 nm TiO_2_, 20 nm Ti, and 200 nm Au through a shadow mask. Polymer thin films were then deposited onto the prepared van der Pauw electrodes. Before AC Hall measurements, an acetone‐soaked cotton applicator was used to gently remove most of the polymer from the contact pads to minimize measurement errors. ≈0.2 mm of polymer was left overlapping each electrode. An input current ranging from 1 nA to 1 mA was applied while the samples were subjected to a magnetic field of 0.5 T, which was modulated at a frequency of 100 mHz. Each measurement was repeated three times, and the reported values represent the averages directly obtained from the system software. An estimated uncertainty of ≈10% was applied to account for measurement errors inherent to the AC Hall setup, mainly due to the systematic phase error of the lock‐in system commonly observed in spin‐coated conjugated polymer films. Additionally, temperature‐dependent measurements of carrier concentration were conducted on both undoped and 3 µg mL^−1^ F_4_TCNQ‐doped P(g_3_2T‐T) samples using the same sample structure. These measurements were taken equipped with a variable temperature cryostat and a turbopump. The Hall voltage was measured at each temperature using a 1.5 T magnetic field modulated at 100 mHz.

### Characterizations—Cyclic Voltammetry (CV) Measurements

CV measurements were conducted using a CH Instruments 600E potentiostat in a standard three‐electrode configuration. The electrochemical cell consisted of a glassy carbon working electrode, an Ag/Ag⁺ reference electrode, and a platinum (Pt) counter electrode. P(g_3_2T‐T) was drop‐casted as a thin film onto the glassy carbon electrode from a hot chloroform solution (1 mg mL^−1^, with tetrabutylammonium hexafluorophosphate added at 100 wt.%). F_4_TCNQ and F_2_TCNQ were measured in solutions at a concentration of 0.5 mg mL^−1^. A 0.1 M solution of tetrabutylammonium hexafluorophosphate in anhydrous acetonitrile served as the supporting electrolyte. The reference electrode was calibrated using a ferrocene/ferrocenium redox couple. All scans were conducted under an argon atmosphere at a scan rate of 100 mV s^−1^.

### Characterizations—Fourier Transform Infrared (FTIR) Spectroscopy

Polymer thin films were deposited on double‐side polished (DSP) silicon substrates for transmission‐mode FTIR measurements. Spectra were collected using a Bruker Hyperion FTIR microscope equipped with a mercury cadmium telluride detector and a Bruker Vertex 70 laser source. After cooling the detector with liquid nitrogen, background correction data were recorded by focusing on a bare DSP silicon substrate. Each film was then centered on the optical path for measurement. Data were acquired in the 600–6000 cm^−1^ range at 4 cm^−1^ resolution and averaged over 64 scans.

### Characterizations—Resonance Raman Spectroscopy

Raman spectra were collected using a Renishaw inVia Raman microscope equipped with a 50× objective in a backscattering configuration. Calibration of the filter and grating was performed using the well‐defined 520 cm^−1^ peak of a silicon reference. Spectra were acquired using a 785 nm laser diode for excitation to probe resonances of P(g_3_2T‐T) and F_4_TCNQ. Acquisition times and laser powers were optimized to give the best spectra and maintained consistently across samples. For each sample, five spectra were collected across the sample area to account for spatial variation, and the mean spectra were presented in the figures. The assignment of P(g_3_2T‐T) Raman peaks was guided by DFT simulations and shows strong agreement with reported spectra for other polythiophenes containing both substituted and unsubstituted thiophene units. The peak at 1444 cm^−1^ and its shoulder at 1460 cm^−1^ correspond to C═C modes of the thiophene rings, with the shoulder more localized to the substituted thiophenes. A prominent C─C mode at 1406 cm^−1^ was used for spectral normalization as it remains relatively unaffected by doping. F_4_TCNQ^−^ peak assignment was based on the simulation and reference to in situ electrochemical Raman spectroscopy data. In this work, two characteristic F_4_TCNQ^−^ peaks were observed at 1453 and 1651 cm^−1^. F_4_TCNQ^2−^ could not be experimentally probed under the conditions employed. Additionally, the tail of the bipolaron band exhibits weak resonance at 785 nm, showing a visible peak at 1505 cm^−1^.

### Characterizations—Density Functional Theory (DFT) Simulations

Raman peak assignments were determined by DFT simulations performed on the Imperial College High‐Performance Computing service using the GAUSSAIN16 software. All simulations were performed on single molecules in the gas phase using B3LYP level of theory and basis set 6–31G(d.p).^[^ ^65^ ^]^ Vibrational frequencies were identified from the simulated Raman spectra using an empirical scaling factor of 0.97. Peak assignments were visualized with the GaussView 6.0.16 software.^[^ ^66^ ^]^

### Characterizations—Space‐Charge Limited Current (SCLC) Measurements

The dark current density‐voltage (*J*‐*V*) characteristics were measured using a Keithley 2400 source meter. To determine the mobilities of both undoped and F_4_TCNQ‐doped P(g_3_2T‐T) (at concentrations of 0.1 and 1 µg mL^−1^), the dark *J−* *V* curves of hole‐only diodes (ITO/PEDOT:PSS/Undoped or doped polymer/MoO_x_/Ag) were fitted to the SCLC model^[^ ^67^ ^]^) according to Mott–Gurney's law, expressed as:

(1) $$J=\frac{9}{8}\;\varepsilon_{r}\varepsilon_{0}\mu_{h}\frac{V^{2}}{L^{3}}$$

where *J* is the current density, *ε*_0_ is the permittivity of free space, *ε*_r_ is the dielectric constant, *µ*_h_ is the hole mobility, *V* is the applied voltage, and *L* is the film thickness.

### Characterizations—Direct‐Current (DC) Hall Measurements

The Ecopia HMS‐3000 Hall measurement system (Ecopia Corporation, Anyang, Gyeonggi‐Do, South Korea) was used to measure the charge carrier mobility through the van der Pauw four‐point probe technique. The sample structure for the measurements was Glass/Polymer/Ag. Input currents varied from 1 nA to 1 mA were applied, and the samples were placed in a magnetic field of 0.55 T.

### Characterizations—Grazing Incidence Wide‐Angle X‐Ray Scattering (GIWAXS) Measurements

GIWAXS measurements were performed at Beamline 7.3.3 of the Advanced Light Source at Berkeley National Laboratory using a Pilatus 2 M area detector. Data were collected at an incident angle of 0.13° and processed with Nika and WAXS tools in Igor Pro. 1D GIWAXS profiles were analyzed using the Multipeak Fitting package.

## Conflict of Interest

The authors declare no conflict of interest.

## Author Contributions

R.S. and J.C. contributed equally to this work. R.S. and F.S. conceived the project. R.S. and J.C. designed the experiments. Sample preparation was carried out by Y.O., J.C., and Y.P. UV‐Vis‐NIR measurements were conducted by J.C. and Y.O., and the analysis of UV‐Vis‐NIR data was performed by R.S. and J.C. Y.O. conducted DC Hall measurements, and SCLC measurements were performed by Y.P., Y.O., and N.C. Y.P., Y.O., and N.C. also conducted film thickness measurements. L.Y. conducted EPR measurements. R.S., J.C., Y.P., and L.Y. contributed to the analysis and interpretation of the EPR data. J.N. synthesized the polymer and performed CV measurements. J.M. performed FTIR measurements under the supervision of A.A., with data analysis and interpretation contributed by J.M., R.S., J.C., and A.A. K.S. carried out Raman characterizations under the supervision of J.‐S.K., with Raman data analysis and interpretation contributed by K.S., R.S., and J.‐S.K. GIWAXS measurements were conducted by S.K. under the supervision of H.A., with analysis and interpretation of the GIWAXS data contributed by S.K., R.S., J.C., and H.A. S.N. and J.H.V. conducted the AC Hall measurements, and R.S., J.C., and Y.P. contributed to the analysis and interpretation of the AC Hall data. W.Y. supervised J.N. and L.Y. R.S. wrote the draft of the paper, which was revised by D.S.G., H.A., W.Y., and F.S. All authors reviewed and commented on the final manuscript.

## Supporting information



Supporting Information

## Data Availability

The data that support the findings of this study are available from the corresponding author upon reasonable request.
