# Supplementary material for: Two‐Stage Bipolaron Formation in Molecularly Doped Conjugated Polymers
Source: Adv Mater. 2025 Aug 6;37(42):e04357. doi: 10.1002/adma.202504357 (PMC12548510; doi:10.1002/adma.202504357)
Supplement: Supplementary file 1 — Supporting Information [file ADMA-37-e04357-s001.docx]

Supporting Information

Two-stage bipolaron formation in molecularly doped conjugated polymers

*Rui Su*, Jingshan Chai, Yusen Pei, Yusuf Olanrewaju, Liang Yan, Justin Neu, Jake Mauthe, Katherine Stewart, Somayeh Kashani, Neha Chaturvedi, Stefan Nikodemski, Jarrett H. Vella, Aram Amassian, David S. Ginger, Ji-Seon Kim, Harald Ade, Wei You, Franky So**

R. Su, J. Chai, Y. Pei, J. Mauthe, N. Chaturvedi, A. Amassian, F. So

Department of Materials Science and Engineering, North Carolina State University, Raleigh, NC 27695, USA

E-mail: rsu3@ncsu.edu, fso@ncsu.edu

Y. Olanrewaju

Department of Fiber and Polymer Science, Wilson College of Textiles, North Carolina State University, Raleigh, NC 27606, USA

L. Yan, J. Neu, W. You

Department of Chemistry, University of North Carolina at Chapel Hill, Chapel Hill, NC 27599, USA

K. Stewart, J.-S. Kim

Department of Physics and Centre for Processable Electronics, Imperial College London, Blackett Laboratory, London, SW7 2AZ, UK

S. Kashani, H. Ade

Department of Physics, North Carolina State University, Raleigh, NC 27695, USA

R. Su, J. Chai, Y. Pei, Y. Olanrewaju, J. Mauthe, S. Kashani, N. Chaturvedi, A. Amassian, H. Ade, F. So

Organic and Carbon Electronics Laboratories (ORaCEL), North Carolina State University, Raleigh, NC 27695, USA

S. Nikodemski, J. H. Vella

Sensors Directorate, Air Force Research Laboratory, Wright-Patterson Air Force Base (AFB), Dayton, OH 45433, USA

D. S. Ginger

Department of Chemistry, University of Washington, Seattle, WA 98195, USA

J.-S. Kim

Department of Chemistry, University of Oxford, Oxford, OX1 3TA, UK

**This file includes the following:**

Figures S1 to S11

Tables S1 to S5

Notes S1 to S5

References


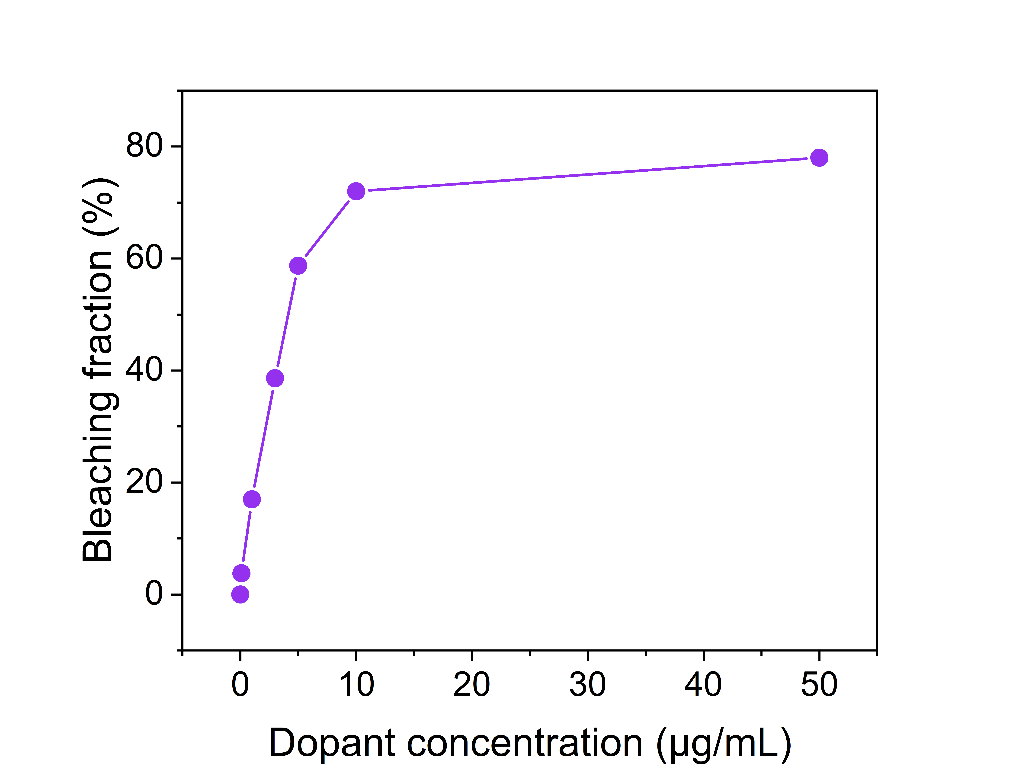


**Figure S1.** Calculated bleaching fraction of P(g_3_2T-T) films dip-doped with F_4_TCNQ.


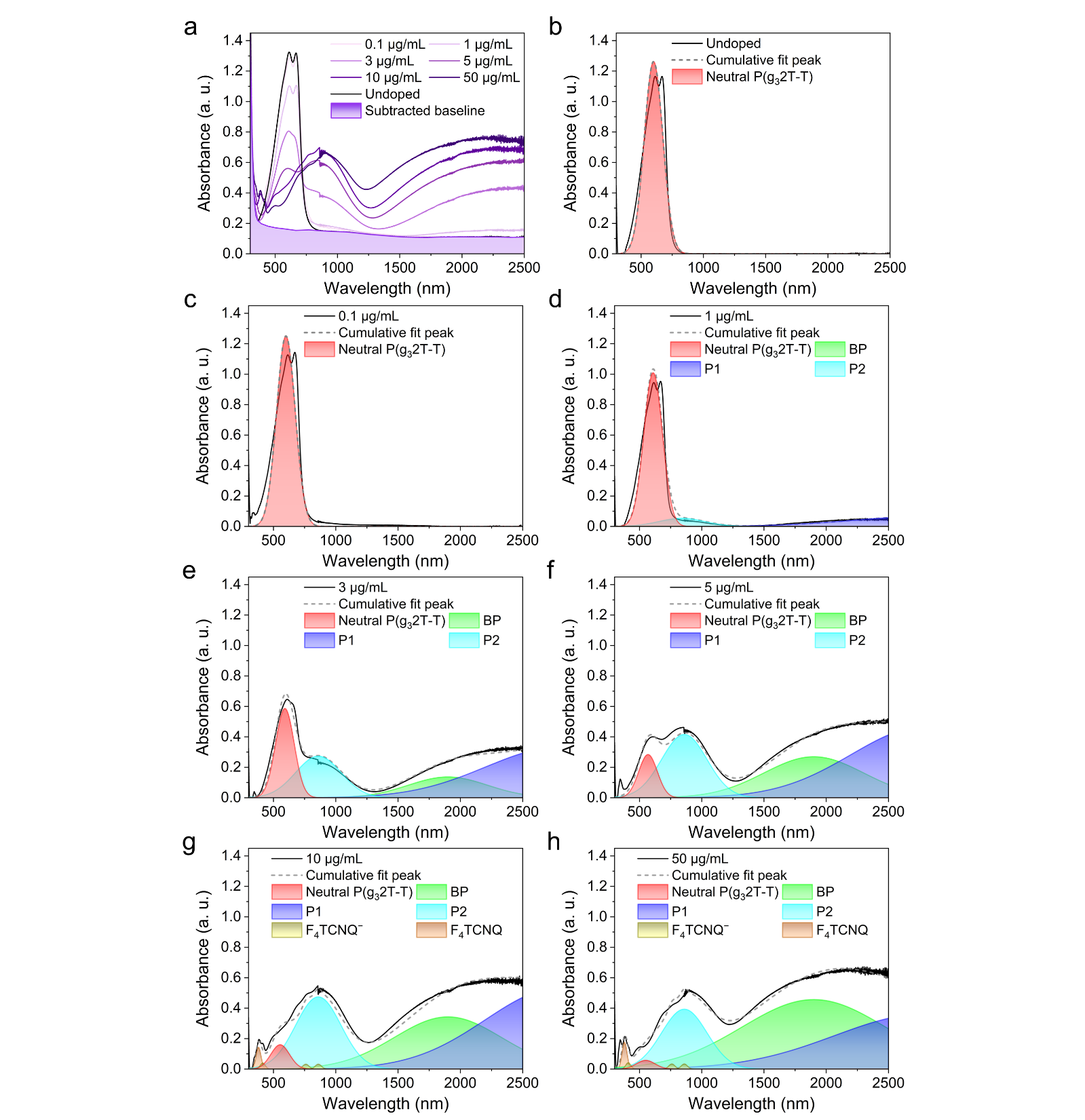


**Figure S2.** Gaussian fitted UV-Vis-NIR absorbance spectra of F_4_TCNQ-doped P(g_3_2T-T) films at varying dopant concentrations. All spectra are baseline-corrected using the undoped film as reference.


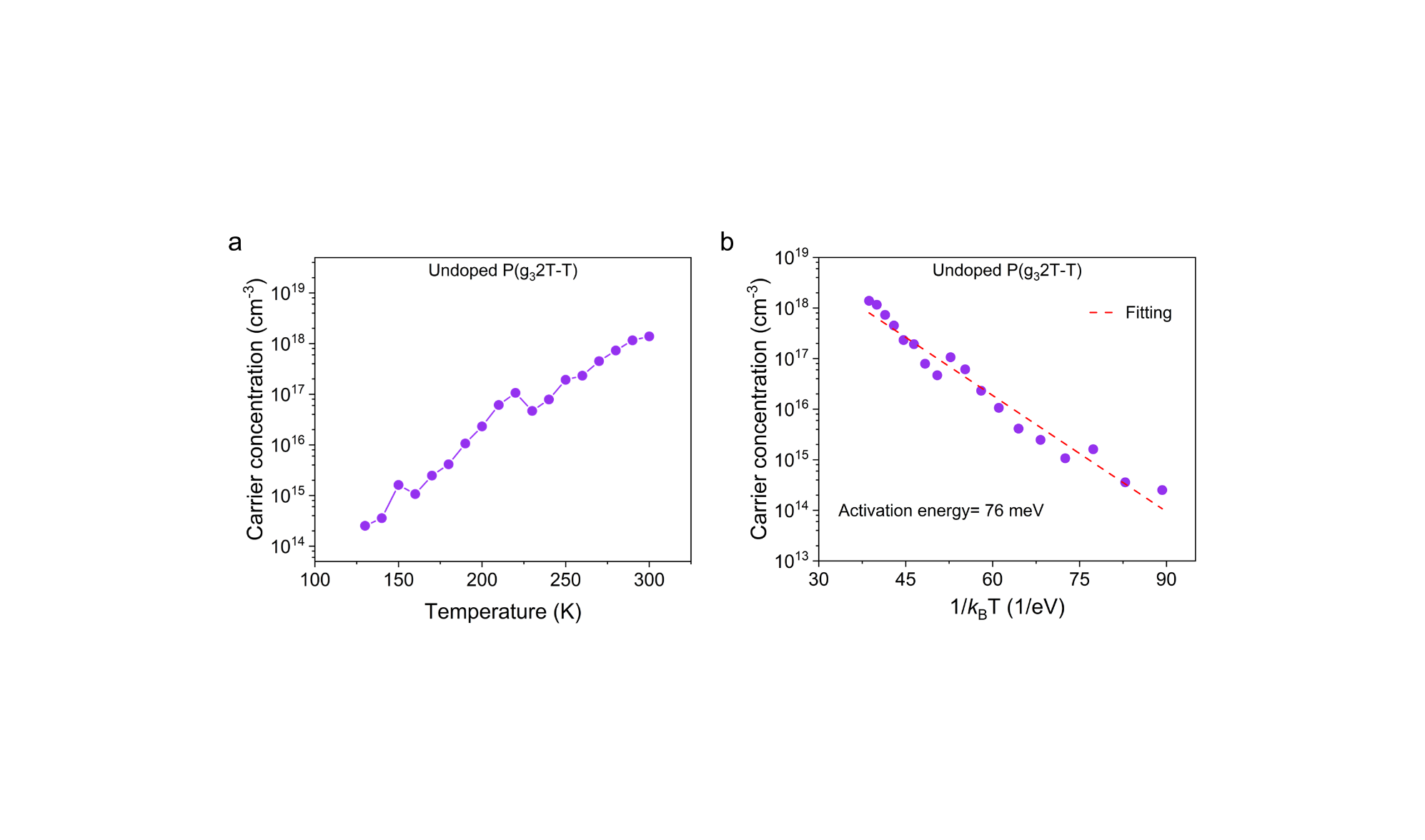


**Figure S3. a**, Carrier concentration of the P(g_3_2T-T) sample as a function of temperature. **b**, Arrhenius plot corresponding to the P(g_3_2T-T) sample. See details in **Note S3**.


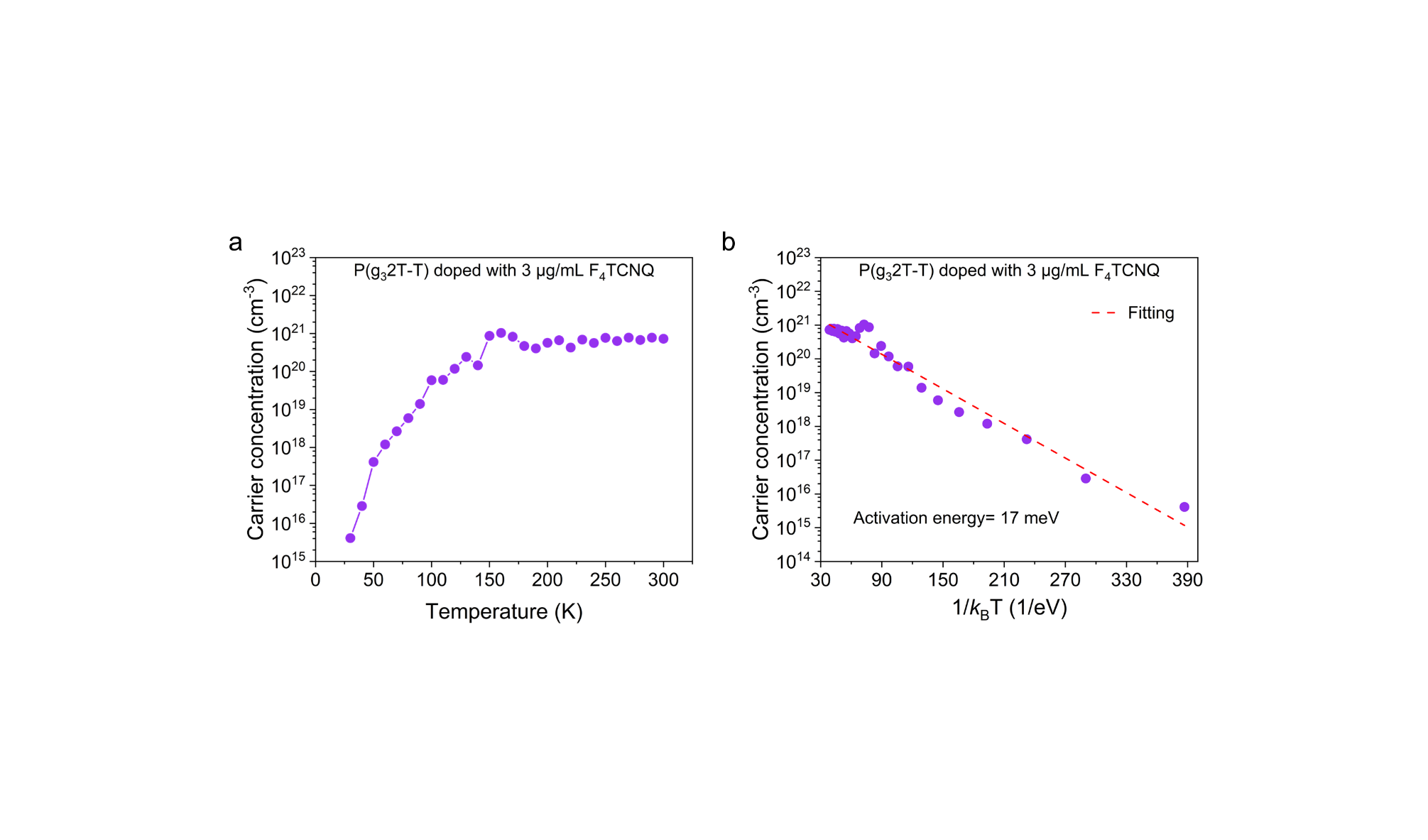


**Figure S4.** **a**, Temperature-dependent carrier concentration of the doped P(g_3_2T-T) sample with 3 µg/mL F_4_TCNQ. **b**, Corresponding Arrhenius plot, with details provided in Note S3.


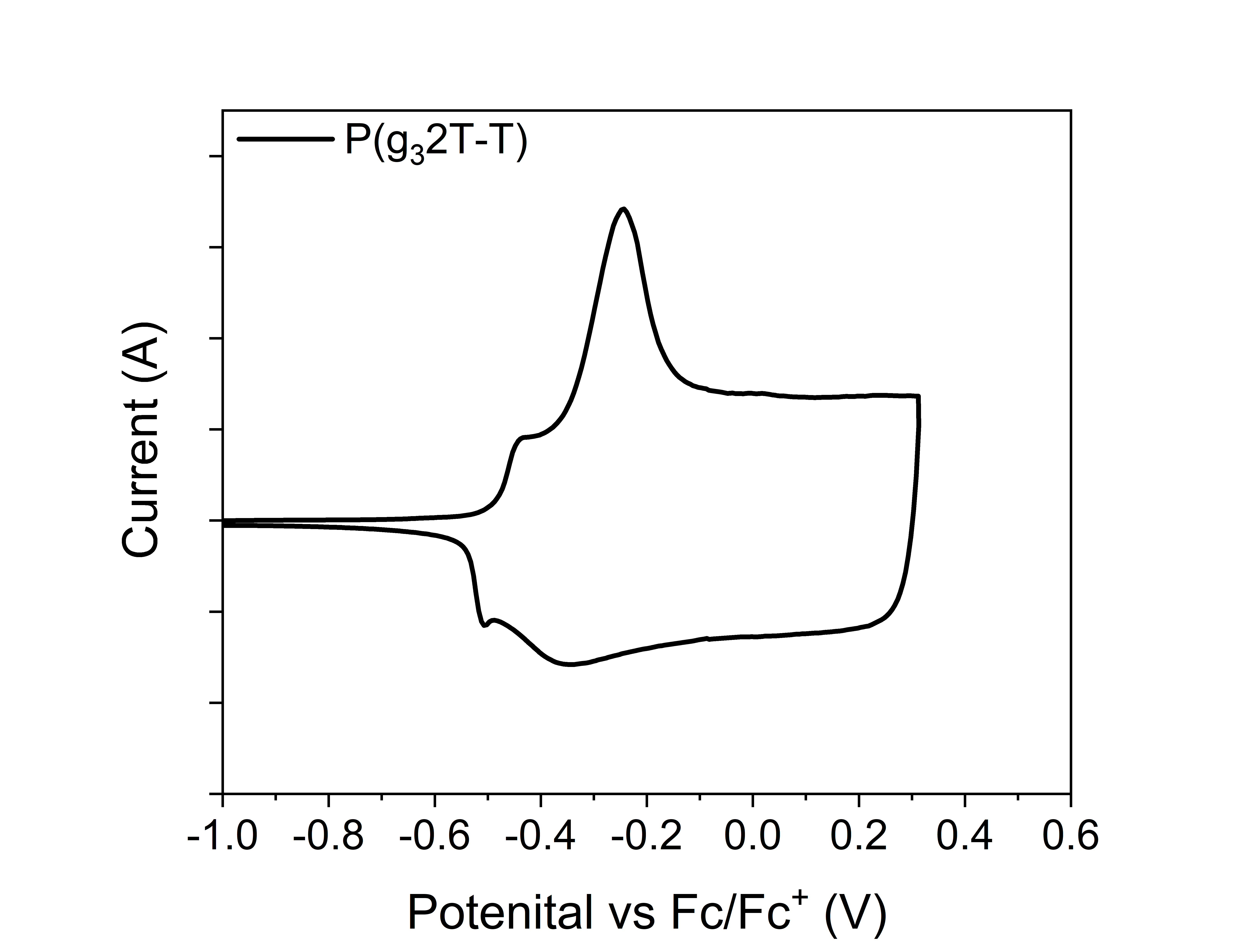


**Figure S5.** The cyclic voltammetry (CV) curve of P(g_3_2T-T).


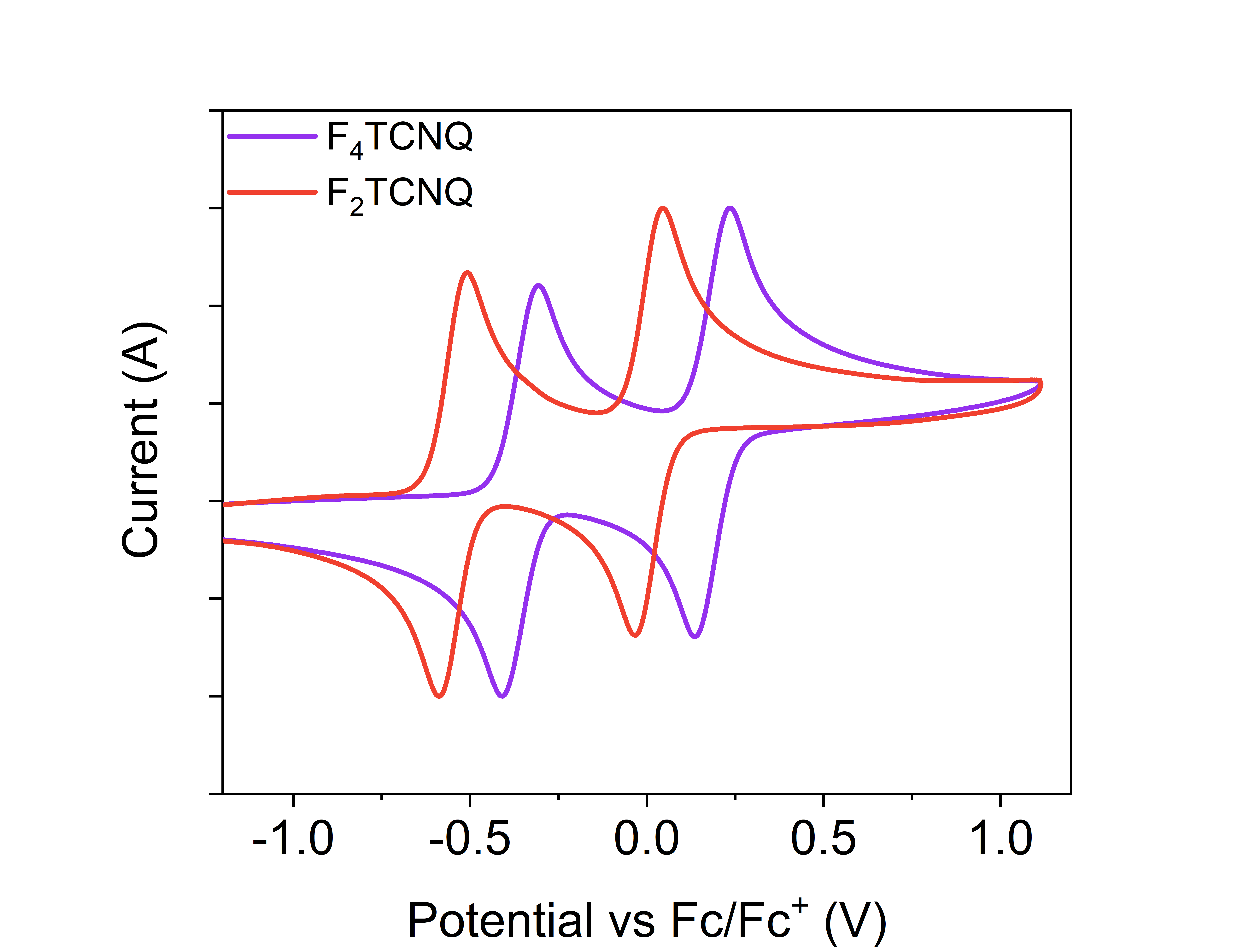


**Figure S6.** CV responses of F_4_TCNQ and F_2_TCNQ.


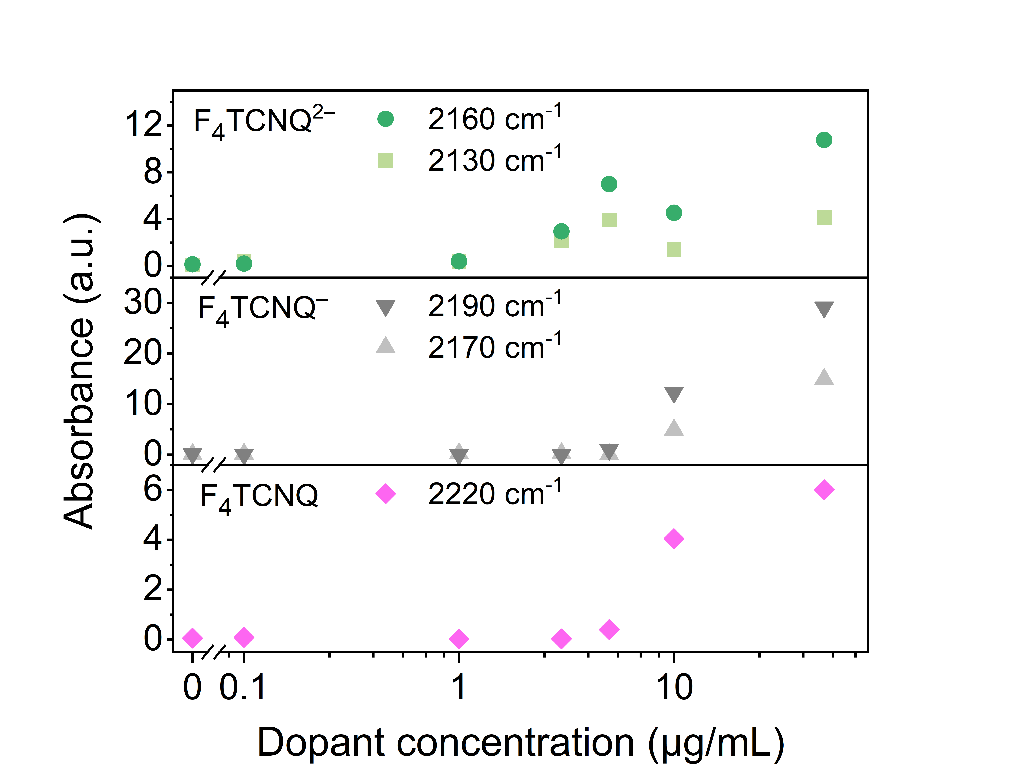


**Figure S7.** Absorbance for the cyano (CN) stretch modes in Fourier transform infrared (FTIR) spectra of F_4_TCNQ^2−^, F_4_TCNQ^−^ and neutral F_4_TCNQ as a function of dopant concentration. The increase in F_4_TCNQ^2−^ peaks at 50 μg/mL can be attributed to overlap with the strong shoulder absorption of F_4_TCNQ^−^.


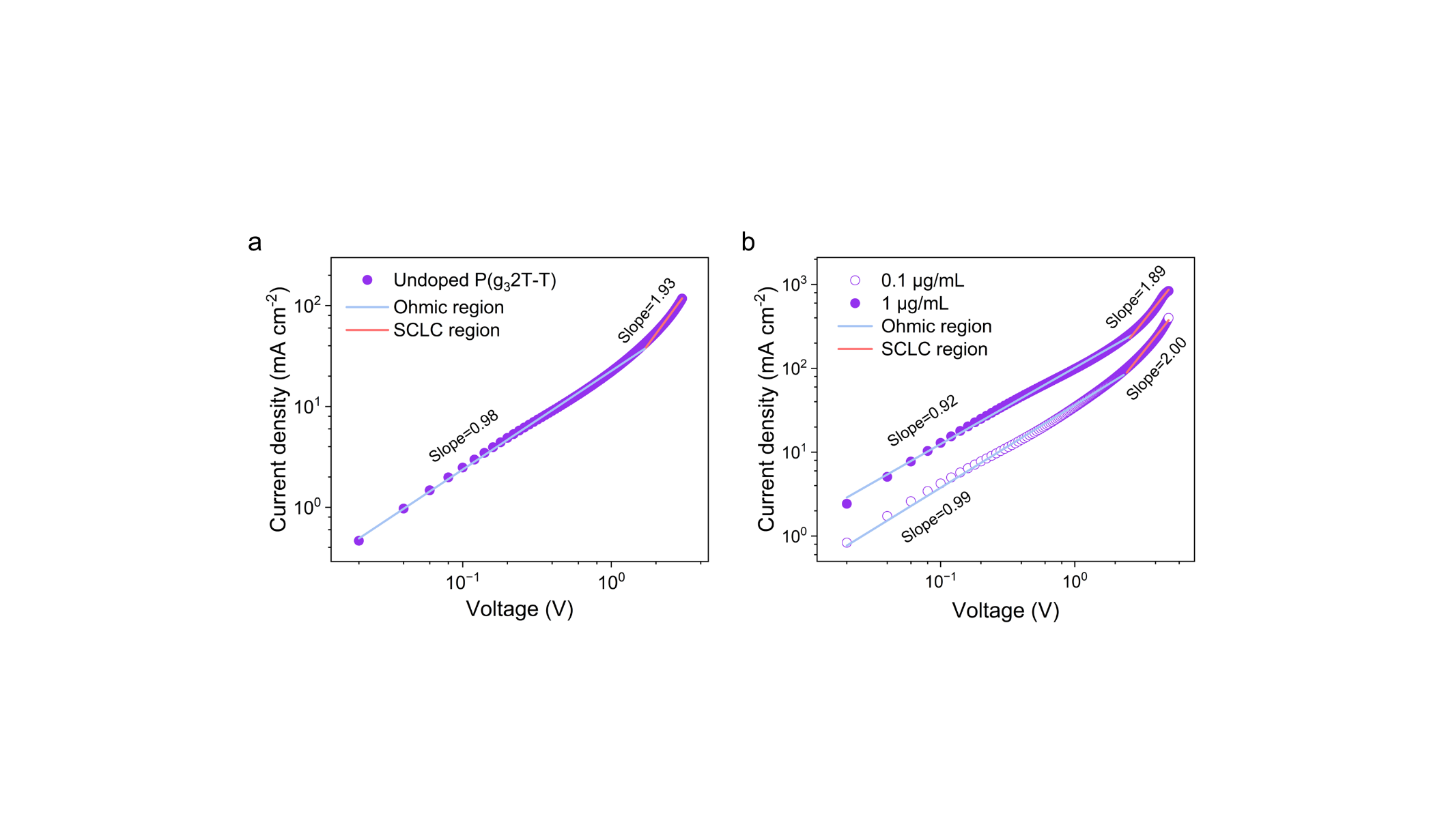


**Figure S8.** Current density-voltage curves obtained from space-charge limited current (SCLC) measurements for **a)** undoped and **b)** F_4_TCNQ-doped P(g_3_2T-T) samples.


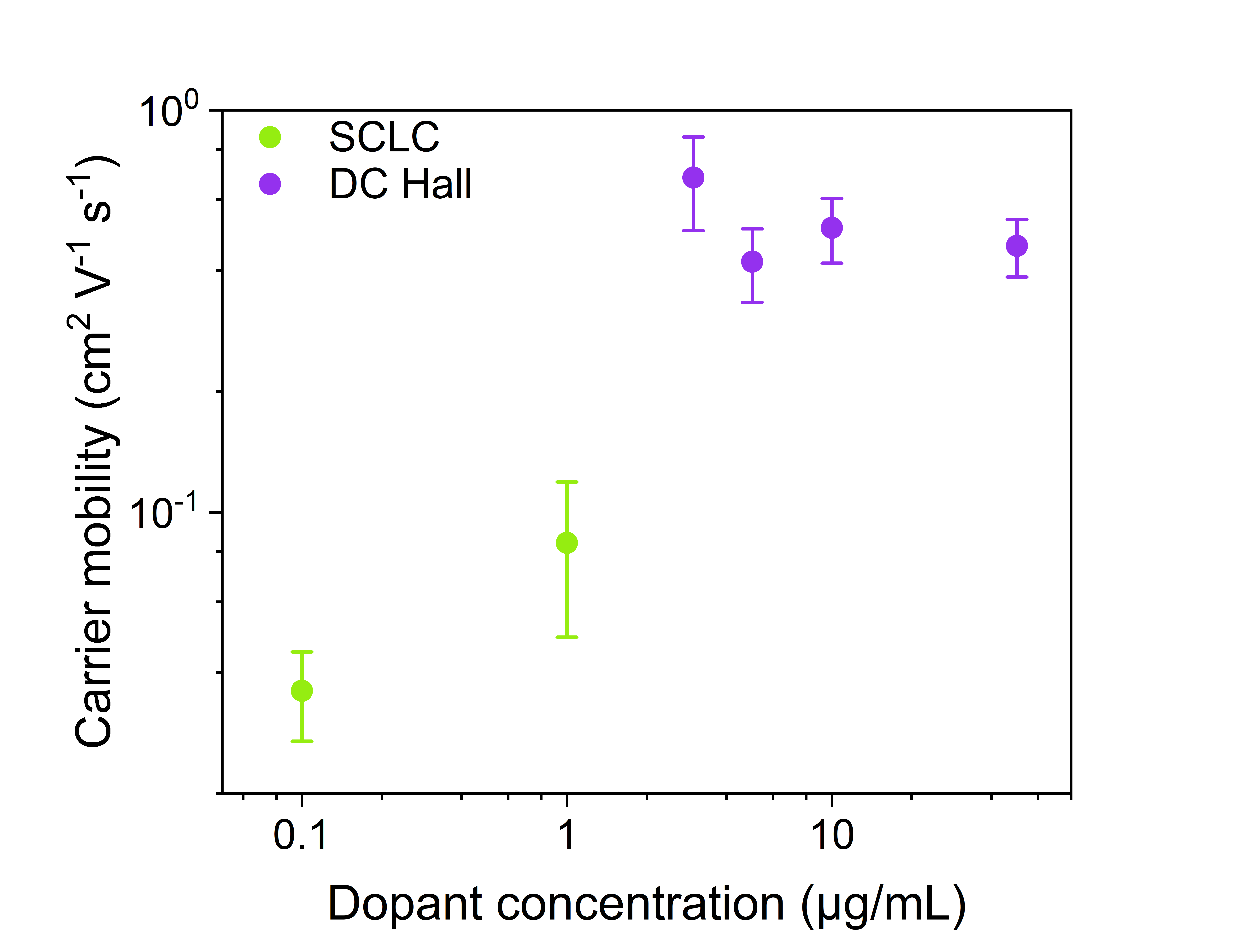


**Figure S9.** Carrier mobility of F_4_TCNQ-doped P(g_3_2T-T) as a function of dopant concentration determined using SCLC and direct-current (DC) Hall measurements.


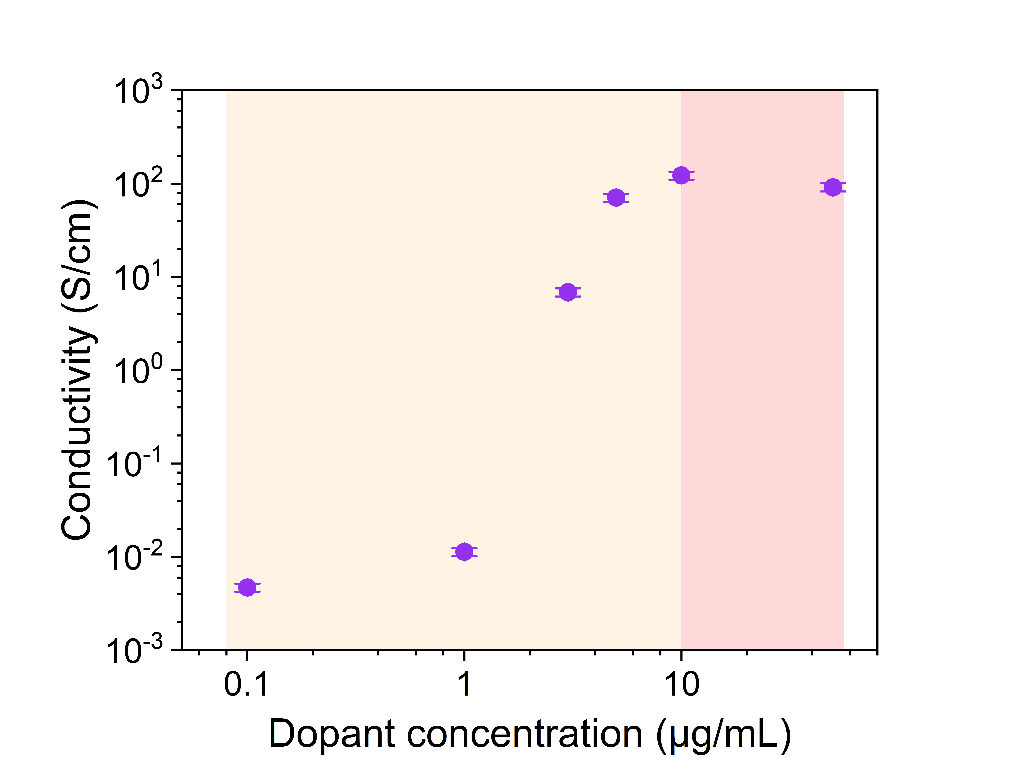


**Figure S10.** Conductivity of F_4_TCNQ-doped P(g_3_2T-T) as a function of dopant concentration by alternating-current (AC) Hall measurements. The two background colors highlight the distinct stages of bipolaron formation at low and high doping levels.


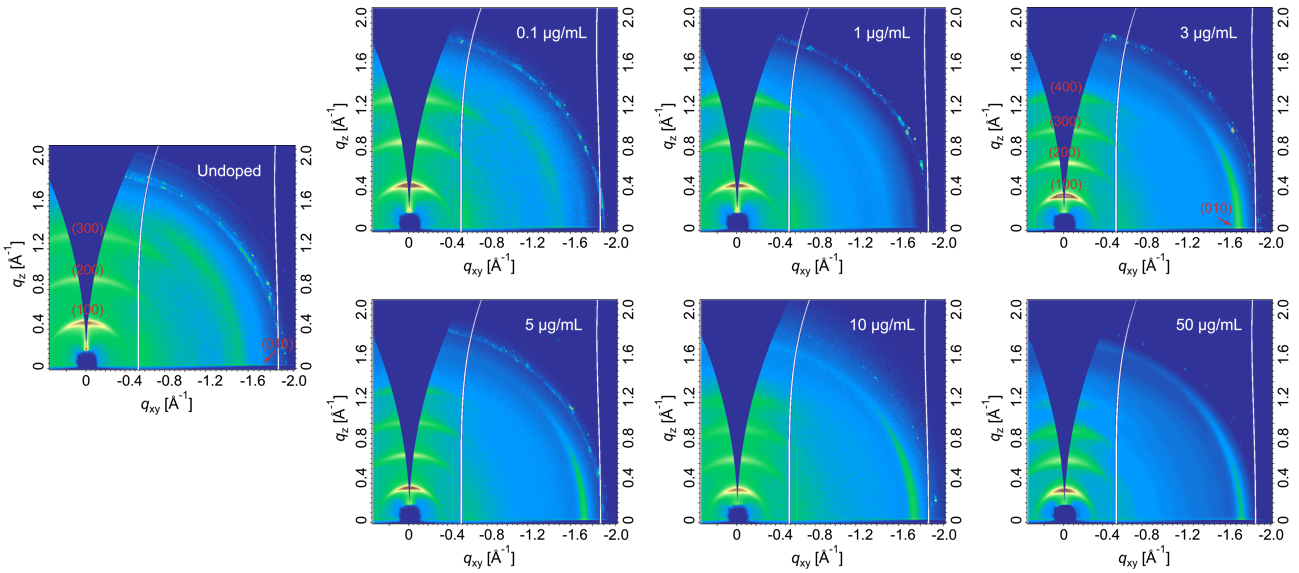


**Figure S11.** Two-dimensional (2D) patterns of grazing incidence wide-angle X-ray scattering (GIWAXS) of P(g_3_2T-T) samples at varying dopant concentrations.

**Table S1.** Spin densities obtained from EPR measurements for three independent sample sets (A–C), along with the standard deviation at each dopant concentration. The spin density values from Set A were used to plot Figure 2d.

| **Dopant concentration**  **(µg/mL)** | **Spin density (cm^-3^)** | | | |
| --- | --- | --- | --- | --- |
|  | **Set A** | **Set B** | **Set C** | **Standard deviation** |
| 0.1 | 2.32×10^19^ | 4.43×10^19^ | 1.91×10^19^ | 1.35×10^19^ |
| 1 | 3.68×10^19^ | 3.08×10^19^ | 3.78×10^19^ | 3.80×10^18^ |
| 3 | 5.73×10^19^ | 7.11×10^19^ | 7.75×10^19^ | 1.04×10^19^ |
| 5 | 7.05×10^19^ | 9.99×10^19^ | 8.85×10^19^ | 1.48×10^19^ |
| 10 | 7.28×10^19^ | 6.11×10^19^ | 6.49×10^19^ | 5.95×10^18^ |
| 50 | 6.41×10^19^ | 1.13×10^20^ | 7.25×10^19^ | 2.61×10^19^ |

**Table S2.** Calculated bipolaron percentages of F_4_TCNQ-doped P(g_3_2T-T) at varying dopant concentrations. See details in Note S4, Supporting Information.

| **Dopant concentration**  **(µg/mL)** | **Carrier concentration**  **(cm^-3^)** | **Spin density**  **(cm^-3^)** | **Bipolaron percentage**  **(%)** |
| --- | --- | --- | --- |
| 0.1 | 2.4 × 10^18^ | 2.3 × 10^19^ | NA^a)^ |
| 1 | 3.6 × 10^18^ | 3.7 × 10^19^ | NA^a)^ |
| 3 | 8.2 × 10^20^ | 5.7 × 10^19^ | 86.9 |
| 5 | 8.7 × 10^20^ | 7.1 × 10^19^ | 85.0 |
| 10 | 1.1 × 10^21^ | 7.3 × 10^19^ | 87.6 |
| 50 | 1.7 × 10^21^ | 6.4 × 10^19^ | 92.7 |

^a)^ A negative bipolaron number is obtained due to the limited mobile carriers at this concentration

**Table S3.** Summary of ionization energy (IE) of the polymer and electron affinity (EA) of the dopants as determined by cyclic voltammetry (CV) measurements.

| **Sample** | **Event** | **Max (V)** | **Min (V)** | ***E*_OX_/*E*_RE_ (V)** | **IE/EA (eV)**^a)^ |
| --- | --- | --- | --- | --- | --- |
| P(g_3_2T-T) | - | -0.46 | -0.52 | -0.49 | 4.60 |
| F_4_TCNQ | 1 | -0.30 | -0.41 | -0.36 | 4.73 |
|  | 2 | 0.23 | 0.13 | 0.18 | 5.27 |
| F_2_TCNQ | 1 | -0.51 | -0.59 | -0.55 | 4.54 |
|  | 2 | 0.04 | -0.03 | 0.01 | 5.10 |

^a)^IE/EA (eV) =[5.09 + *E* (V)] eV

**Table S4.** Molar-to-mass concentration conversions of F_4_TCNQ and F_2_TCNQ solutions for sequential doping.

| **Molar concentration**  **(μmol/mL)** | **Mass concentration** | |
| --- | --- | --- |
|  | **F_4_TCNQ (μg/mL)** | **F_2_TCNQ (μg/mL)** |
| 3.62 × 10^-4^ | 0.1 | 0.087 |
| 3.62 × 10^-4^ | 1 | 0.87 |
| 0.0109 | 3 | 2.61 |
| 0.0181 | 5 | 4.35 |
| 0.0362 | 10 | 8.70 |
| 0.0724 | 20 | 17.4 |
| 0.109 | 30 | 26.1 |
| 0.145 | 40 | 34.8 |
| 0.181 | 50 | 43.5 |
| 0.362 | 100 | 87.0 |

**Table S5.** Bleaching fractions of P(g_3_2T-T) films sequentially doped with F_4_TCNQ and F_2_TCNQ.

| **F_4_TCNQ-doped** | | **F_2_TCNQ-doped** | |
| --- | --- | --- | --- |
| **Concentration (μg/mL)** | **Bleaching fraction (%)** | **Concentration (μg/mL)** | **Bleaching fraction (%)** |
| 0.1 | 3.9 | 0.087 | 2.4 |
| 1 | 6.2 | 0.87 | 4.6 |
| 3 | 13.9 | 2.61 | 9.6 |
| 5 | 18.6 | 4.35 | 10.7 |
| 10 | 24.8 | 8.70 | 16.4 |
| 20 | 32.5 | 17.4 | 21.2 |
| 30 | 44.9 | 26.1 | 26.4 |
| 40 | 54.3 | 34.8 | 43.3 |
| 50 | 58.1 | 43.5 | 55.8 |
| 100 | 62.8 | 87.0 | 58.6 |

**Note S1.** Synthesis of poly[5,5'-(3,3-bis(2-(2-(2-methoxyethoxy)ethoxy)ethoxy)-2,2'-bithiophene)-alt-2,5-thiophene]

Synthesis of monomer of P(g_3_2T-T)

5,5'-dibromo-3,3'-bis(2-(2-(2-methoxyethoxy)ethoxy)ethoxy)-2,2'-bithiophene was synthesized according to previous literature.^[1]^ ^1^H NMR (400 MHz, CDCl_3_) δ 6.85 (s, 1H), 4.19 (dd, *J* = 5.7, 4.0 Hz, 2H), 3.90 – 3.83 (m, 2H), 3.77 – 3.71 (m, 2H), 3.71 – 3.64 (m, 4H), 3.59 –3.52 (m, 2H), 3.38 (s, 3H).


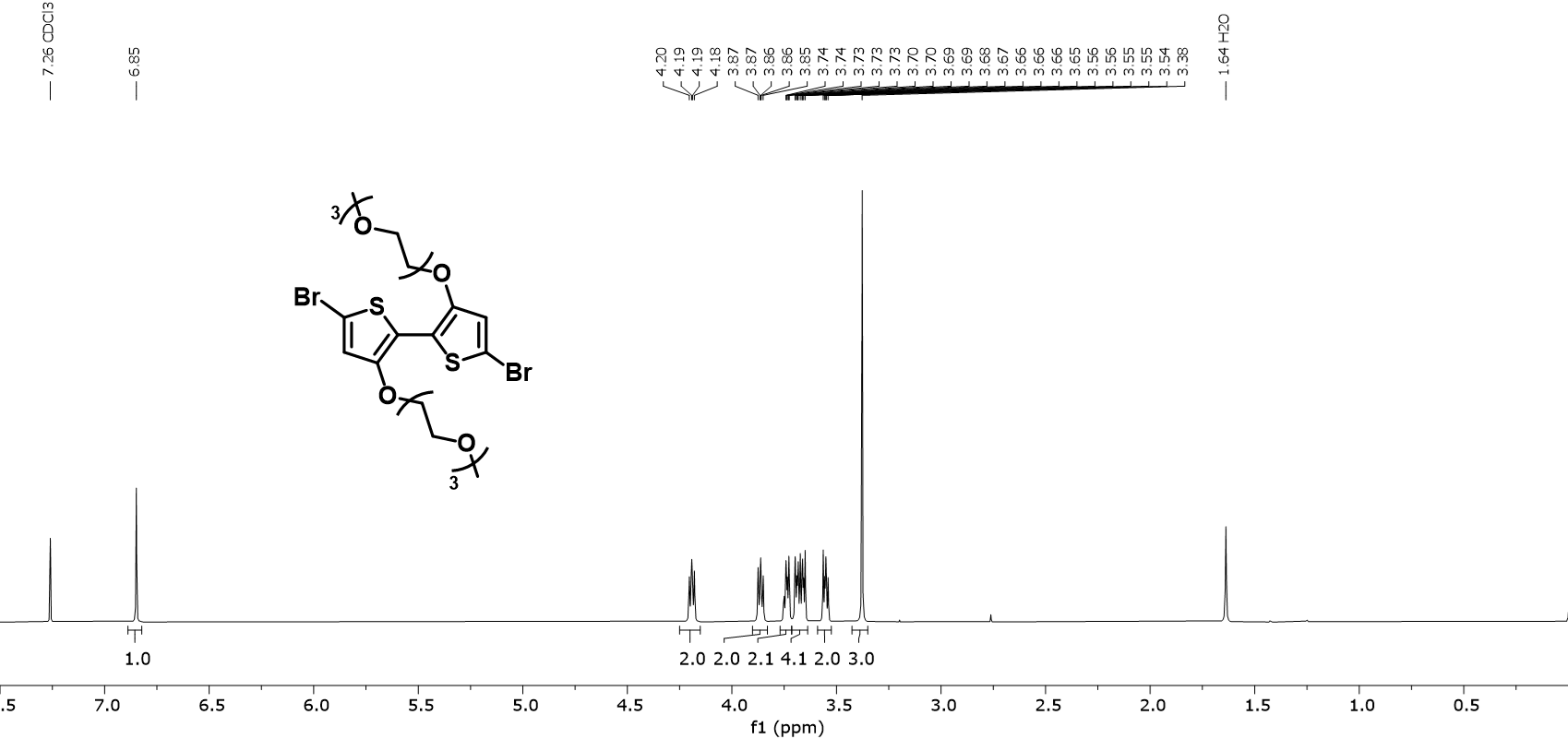


Polymerization of P(g_3_2T-T)

195.91 mg (1 equivalent, 302.13 μmol) of 5,5'-dibromo-3,3'-bis(2-(2-(2-methoxyethoxy)ethoxy)ethoxy)-2,2'-bithiophene, 123.80 mg (1.00 equivalent, 302.13 μmol) of 2,5-bis(trimethylstannyl)thiophene, and 13.966 mg (0.04 equivalent, 12.085 μmol) of tetrakis(triphenylphosphine)palladium(0) were combined in a 10 mL microwave reactor vial. The mixture was degassed with argon (three evacuation-refill cycles) before adding anhydrous toluene (2.2 mL) and DMF (0.22 mL) under an argon flow. The reaction was heated to 200 °C for 30 minutes in a CEM microwave reactor (300 W). Following polymerization, the crude polymer was dissolved in hot tetrachloroethane, precipitated in hexanes, and then extracted with ethyl acetate using a Soxhlet extractor. The collected polymer was concentrated in chloroform, re-precipitated, and isolated via filtration. Finally, the polymer was dried under vacuum, yielding a dark solid (123 mg). Its molecular weight was measured using gel permeation chromatography (GPC) in dimethylformamide (DMF) at 55 °C, as shown below, resulting in a number average molecular weight (*M*_n_) of ~33 kDa, a weight average molecular weight (*M*_w_) of ~49 kDa, and a dispersity (*Đ*) of ~1.5. Note: significant aggregation prevents accurate molecular weight characterization.


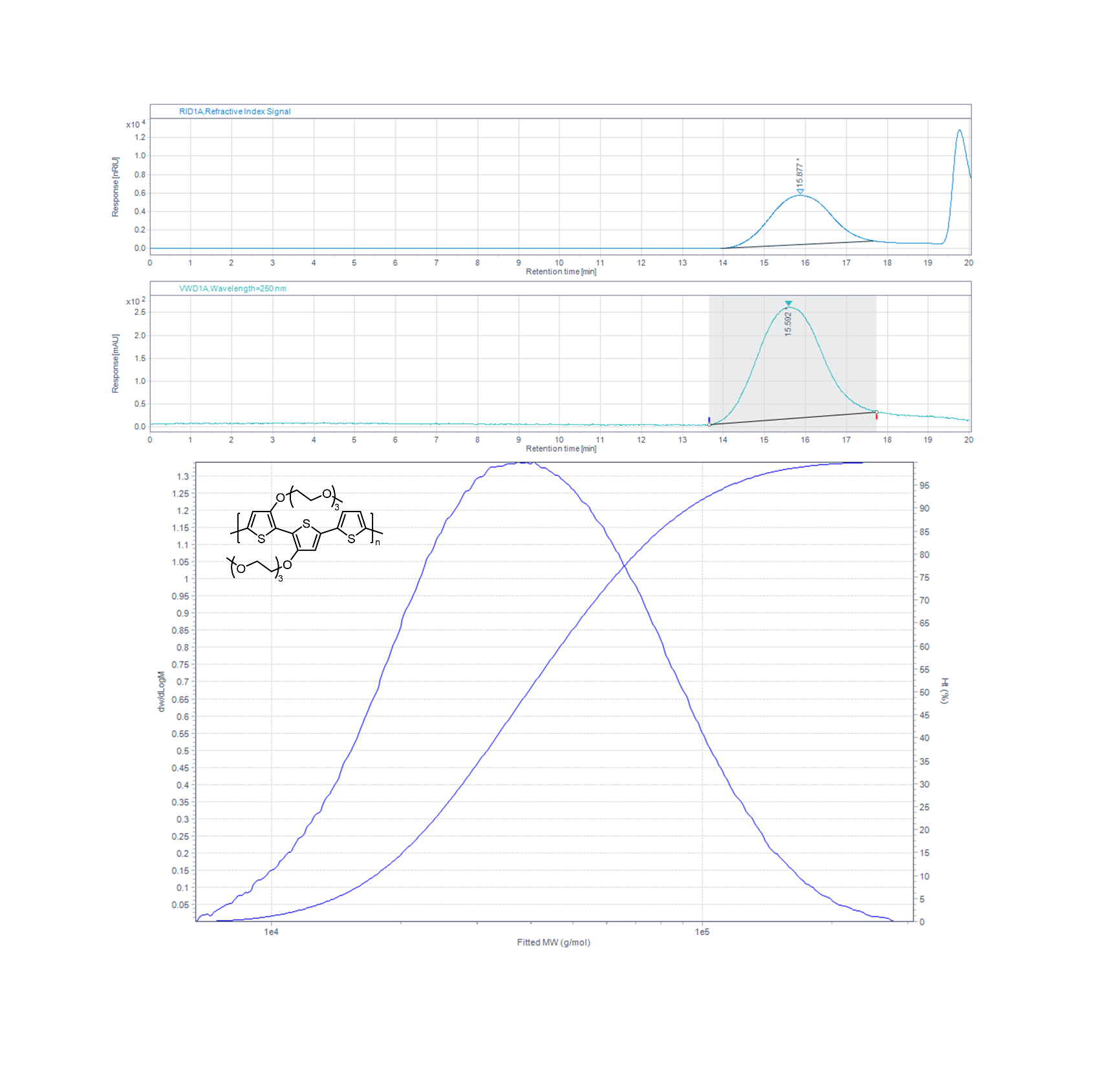


**Note S2.** Bleaching fraction calculation

The bleaching fraction of P(g_3_2T-T) films doped with F_4_TCNQ was determined by analyzing the UV-Vis-NIR absorbance spectra. This fraction reflects the reduction in the intensity of the π−π* absorption peak characteristic of the polymer upon doping, which provides a measure of the extent of charge transfer between the polymer and the dopant. The bleaching fraction was quantified using the following equation:

$Bleaching fraction (\%)= \left( \frac{A_{0}-A_{\mathrm{doped}}}{A_{0}} \right)\times100$ (S1)

where *A*_0_ represents the absorbance of undoped P(g_3_2T-T) at the characteristic peak, and *A*_doped_ is the absorbance at the same wavelength after doping.

**Note S3.** Extraction of activation energy *E*_a_

The activation energy (*E*_a​_) is crucial for understanding the charge carrier dynamics by analyzing the temperature-dependent behaviors in doping systems where thermally activated transport dominates.^[2]^ The *E*_a​_ values for both undoped and doped samples (3 µg/mL) were extracted from temperature-dependent AC Hall measurements across the entire measurement range as shown in Figure S3 and S4, Supporting Information. The extraction was obtained according to the Arrhenius equation:^[3]^

$n= n_{0}\exp\left( \frac{E_{a}}{k_{B}T} \right)$ (S2)

where *n* denotes the carrier concentration, *n*_0_ is a temperature-independent perfector, *k*_B_*​* is the Boltzmann constant, and *T* is the absolute temperature.

**Note S4.** Estimation of bipolaron percentage

The bipolaron percentage is calculated as:

$\mathrm{Bipolaron} \mathrm{percentage} \left( \% \right)=\frac{N_{\mathrm{bipolaron}^{2+}}}{N_{\mathrm{bipolaron}^{2+}}+ N_{\mathrm{polaron}^{+}}} \times100$ (S3)

where $\text{N}_{\text{bipolaron}^{\text{2+}}}$ and $\text{N}_{\text{polaron}^{\text{+}}}$ are the number of bipolarons and polarons, respectively, defined as:

$N_{\mathrm{bipolaron}^{2+}}=c_{\mathrm{bipolaron}^{2+}}\times V$ (S4)

$N_{\mathrm{polaron}^{+}}=c_{\mathrm{polaron}^{+}}\times V$ (S5)

Here, $\text{c}_{\text{bipolaron}^{\text{2+}}}$ and $\text{c}_{\text{polaron}^{\text{+}}}$ represent the concentrations of bipolarons and polarons, and *V* is the volume for the test sample.

Since each polaron carries one unpaired spin, the polaron concentration can be estimated from the spin density measured by EPR:

$c_{\mathrm{spin}}=c_{\mathrm{polaro}n^{+}+}c_{F_{4}\mathrm{TCN}Q^{-}}$ (S6)

where *c*_spin_ is the total spin density, and $\text{c}_{\text{F}_{\text{4}}\text{TCN}\text{Q}^{\text{-}}}$ represents the concentration of F_4_TCNQ⁻, which also carry one unpaired spin and may contribute to the measured signal in our system.

Given the absence of the characteristic F_4_TCNQ⁻ absorption peaks at 765 nm and 862 nm^[4]^ in the UV-Vis-NIR spectra at low doping levels (Figure 2a), the contribution from F_4_TCNQ⁻ can be considered negligible at these dopant concentrations (≤ 5 μg/mL). Therefore, the following approximation can be made:

$c_{\mathrm{polaro}n^{+}}{\approx c}_{\mathrm{spin}}$ (S7)

Since bipolarons are spinless species, the concentration of bipolarons can be derived from the total carrier concentration *c*_carrier_, obtained from AC Hall measurements:

$c_{\mathrm{carrier}}= {2\times c}_{\mathrm{bipolaro}n^{2+}}+c_{\mathrm{polaro}n^{+}}$ (S8)

Substituting Equation S7 into S8 gives the bipolaron concentration:

$c_{\mathrm{bipolaro}n^{2+}}=\frac{{(c}_{\mathrm{carrier}}-c_{\mathrm{spin}})}{2}$ (S9)

Finally, combining Equations S3-S5, S7, and S9, the bipolaron percentage simplifies to:

$Bipolaron percentage \left( \% \right)=\frac{c_{\mathrm{carrier}}-c_{\mathrm{spin}}}{c_{\mathrm{carrier}}+c_{\mathrm{spin}}}\times100$ (S10)

This expression enables a direct estimation of the bipolaron percentage based on the measured spin density and carrier concentration, assuming negligible contributions from F_4_TCNQ⁻ to the spin density. At high doping levels (10 μg/mL and 50 μg/mL), however, the calculated bipolaron percentages may be underestimated due to additional spin contributions from F_4_TCNQ⁻.

**Note S5.** Definition for the analysis of GIWAXS

1. Interplanar spacing *d_hkl_*

$d_{hkl}=\frac{2\pi}{q_{hkl}}$ (S11)

2. Standard deviation of lattice spacing *∆q_hkl_*

$\Delta q_{hkl}=$FWHM (S12)

where FWHM is the full width at half maximum of the peak.

3. Coherence length (*L*_c_) denotes the average size of crystalline domains

$L_{c} =\frac{2\pi K}{\Delta q_{hkl}}$ (S13)

where *K* is a shape factor and is usually equal to 0.9.

4. Paracrystallinity parameter (*g*) describes the overall lattice disorder within an imperfect crystal and is defined as the standard deviation of lattice spacing distributions normalized by the average lattice spacing

$g =\frac{1}{2\pi}\sqrt{\Delta q_{hkl}\times d_{hkl}}$ (S14)

Here, *L*_c_ and *g* across different doping levels can assist in quantifying the structural disorder induced by doping.

**References**

[1] R. Kroon, D. Kiefer, D. Stegerer, et al., "Polar Side Chains Enhance Processability, Electrical Conductivity, and Thermal Stability of a Molecularly p-Doped Polythiophene", *Advanced Materials* 29, no. 24 (2017): 1700930. <https://doi.org/10.1002/adma.201700930>

[2] K. Yamada, B. Shinozaki, T. Narikiyo, et al., "Temperature Dependence of Mobility of Conducting Polymer Polyaniline with Secondary Dopant", *Synthetic Metals* 247, (2019): 124. <https://doi.org/10.1016/j.synthmet.2018.11.019>

[3] P. Boolchand, *Insulating and Semiconducting Glasses.* (WORLD SCIENTIFIC, 2000), ISBN 9789810236731.

[4] S.-J. Kwon, R. Giridharagopal, J. Neu, et al., "Quantifying Doping Efficiency to Probe the Effects of Nanoscale Morphology and Solvent Swelling in Molecular Doping of Conjugated Polymers", *The Journal of Physical Chemistry C* 128, no. 6 (2024): 2748. <https://doi.org/10.1021/acs.jpcc.4c00153>
